# NF-κB directly mediates epigenetic deregulation of common microRNAs in Epstein-Barr virus-mediated transformation of B-cells and in lymphomas

**DOI:** 10.1093/nar/gku826

**Published:** 2014-09-08

**Authors:** Roser Vento-Tormo, Javier Rodríguez-Ubreva, Lorena Di Lisio, Abul B. M. M. K. Islam, Jose M. Urquiza, Henar Hernando, Nuria López-Bigas, Claire Shannon-Lowe, Nerea Martínez, Santiago Montes-Moreno, Miguel A. Piris, Esteban Ballestar

**Affiliations:** 1 Chromatin and Disease Group, Cancer Epigenetics and Biology Programme (PEBC), Bellvitge Biomedical Research Institute (IDIBELL), 08908 L'Hospitalet de Llobregat, Barcelona, Spain; 2 Pathology Department, Hospital Universitario Marques de Valdecilla, Cancer Genomics, IFIMAV, 39008 Santander, Spain; 3 Department of Genetic Engineering and Biotechnology, University of Dhaka, Dhaka 1000, Bangladesh; 4 Department of Experimental and Health Sciences, Barcelona Biomedical Research Park, Universitat Pompeu Fabra (UPF), 08003 Barcelona, Spain; 5 Catalan Institution for Research and Advanced Studies (ICREA), 08003 Barcelona, Spain; 6 CR-UK Institute for Cancer Studies, University of Birmingham, Birmingham B15 2TT, UK

## Abstract

MicroRNAs (miRNAs) have negative effects on gene expression and are major players in cell function in normal and pathological conditions. Epstein-Barr virus (EBV) infection of resting B lymphocytes results in their growth transformation and associates with different B cell lymphomas. EBV-mediated B cell transformation involves large changes in gene expression, including cellular miRNAs. We performed miRNA expression analysis in growth transformation of EBV-infected B cells. We observed predominant downregulation of miRNAs and upregulation of a few miRNAs. We observed similar profiles of miRNA expression in B cells stimulated with CD40L/IL-4, and those infected with EBNA-2- and LMP-1-deficient EBV particles, suggesting the implication of the NF-kB pathway, common to all four situations. In fact, the NF-kB subunit p65 associates with the transcription start site (TSS) of both upregulated and downregulated miRNAs following EBV infection This occurs together with changes at histone H3K27me3 and histone H3K4me3. Inhibition of the NF-kB pathway impairs changes in miRNA expression, NF-kB binding and changes at the above histone modifications near the TSS of these miRNA genes. Changes in expression of these miRNAs also occurred in diffuse large B cell lymphomas (DLBCL), which are strongly NF-kB dependent. Our results highlight the relevance of the NF-kB pathway in epigenetically mediated miRNA control in B cell transformation and DLBCL.

## INTRODUCTION

The Epstein-Barr virus (EBV) is one of the best studied oncogenic human herpesvirus. The vast majority of the human population is infected by EBV. Fortunately, the most common pattern of EBV infection is a clinically silent childhood infection and, generally, EBV establishes a permanent latent infection without further complications. However, EBV has oncogenic potential, reflected by its ability to growth transform B lymphocytes *in vivo*. EBV is associated with different tumors, including several B cell lymphomas, and carcinomas of the stomach and the nasopharyngeal cavity. EBV is considered to play a causative role in Burkitt lymphomas although the mechanism remains elusive (1). EBV has a less strict association with Hodgkin's lymphoma and diffuse large B cell lymphoma (DLBCL). However, the great prevalence of these latter two lymphoma types makes the study of the changes associated with EBV infection of B-cells and biology particularly relevant to our understanding of lymphoma pathogenesis.

*In vitro*, EBV efficiently immortalizes primary resting B lymphocytes (RBLs), converting them into permanently growing lymphoblastoid cell lines (LCLs). *In vitro* infection results in the activation of a specific viral gene expression program that involves expression of six nuclear antigens (EBNA-1, -2, 3A, -3B, -3C and -LP), three membrane proteins (LMP-1, -2A and -2B) and a set of 25 microRNAs (miRNAs). Five of these proteins and several of the miRNAs are essential for transformation. For instance, LMP-1 is required to establish cell transformation *in vitro* (2) and is required for continuous proliferation (3). It has also been reported that members of the EBV miRNA cluster cooperate to transform B lymphocytes (4). Infection of B cells with EBV is similar to the physiological stimulation with CD40L plus IL-4 (5), T cell-derived mitogens and in both cases involves the activation of the NF-kB pathway. Dissection of the cell pathways has shown that EBV can also make use of the NF-kB pathway through LMP-2A in EBV-associated epithelial carcinoma (6) and it is likely that LMP-2A could have similar effects following infection of B cells.

EBV-mediated growth-transformation of B cells results in major changes in gene expression and nuclear reorganization. Changes in gene expression levels depend on a variety of mechanisms including not only transcription factor-mediated and epigenetic control (7) but also post-transcriptional regulation, such as those dependent on viral but also cellular miRNAs. We and other researchers have previously investigated the effects of experimental infection with EBV on epigenetic marks. For instance, EBV infection leads to demethylation of genes within the B cell transcription program (8) and contributes to the overexpression of genes essential for transformation. Also, analyses of histone modifications have shown that EBV infection results in both global and gene-specific changes in different modifications, which also contribute to key changes in gene expression during the growth-transformation of B cells (9).

MicroRNAs are a class of non-coding genes with broad influences on cellular signal transduction pathways. They function by inhibiting translation of select groups of mRNA transcripts containing imperfect annealing sequences in their 3′ untranslated regions (3′ UTRs) and, less frequently, through other regions of the transcript. Previous studies have shown that EBV infection results in upregulation of several miRNAs. For instance, miR-34a is strongly induced by EBV (10) and is associated with growth promotion. It has also been demonstrated that miR-155 is upregulated following EBV infection. These miRNAs are also strongly upregulated in B cell lymphomas (11,12) and it has been proposed that miRNAs misregulation in lymphomas could be used for diagnosis, prognosis or prediction of response to specific therapies (13). As aforementioned, DLBCL is one of the B cell lymphoma types associated with EBV (14) and also the most common type of lymphoma, accounting for 30–40% of lymphomas in western countries. On the basis of the correlation between microarray gene expression profiling and clinical outcome, it is now possible to classify the majority of DLBCLs into molecular variants called activated B cell-like DLBCL (ABC-DLBCL) and germinal center-like DLBCL (GC-DLBCL). A distinguishing feature of DLBCL is a signature of genes that are induced by NF-kB, which is upregulated in ABC-DLBCL (15) but not in GC-DLBCL.

We currently know little about the overall relevance of miRNAs in EBV-mediated transformation of B cells and about how much of the miRNA expression footprint in B cell lymphomas is associated with EBV primary infection. In this study, we investigated the deregulation of human miRNAs during EBV-mediated transformation of RBLs to LCLs, using a high-throughput strategy. We observed significant upregulation of several miRNAs, although miRNA downregulation was highly predominant. Time-course analysis indicated that miRNA deregulation occurs before cell proliferation, particularly for those that become upregulated. Also, analysis of B cells infected with EBV deficient in EBNA-2 and LMP-1, and with B cells stimulated with IL-4/CD40L, revealed that similar changes in miRNA expression occur, suggesting a potential role of the stimulation of the NF-kB pathway that is common to all these conditions. We showed that changes in miRNA expression occur in parallel with changes in histone H3K4me3 and H3K27me3. The involvement of NF-kB in miRNA deregulation was demonstrated by the enrichment of the binding motifs of the NF-kB complex subunits among the genomic sequences of the miRNAs undergoing expression changes, NF-kB p65 binding to miRNA promoters from ChIP-seq data and the finding that p65 binds the promoters of miRNAs following EBV infection. Moreover, we showed that the use of NF-kB inhibitors impairs the expression changes of these miRNAs, and abrogates both the association of the NF-kB subunit p65 as well as changes in histone modifications near the transcription start site (TSS) of these miRNAs. We also observed a large overlap in miRNA expression changes when comparing the profiles associated with EBV-mediated transformation of B cells with the profiles obtained for DLBCL primary samples. miRNA expression changes are related to changes in the levels of targets relevant to B cell transformation and in DLBCL. Our findings identify a novel mechanistic link between NF-kB and epigenetic regulation of miRNAs in EBV-mediated transformation of B cells and their implications in this model and in DLBCLs.

## MATERIALS AND METHODS

### Ethics statement

Human blood samples used in this study came from anonymous blood donors and were obtained from the Catalan blood donation center (Banc de Sang i Teixits). The anonymous blood donors received oral and written information about the possibility that their blood would be used for research purposes, and any questions that arose were then answered. Before giving their first blood sample the donors signed a consent form at the Banc de Teixits, which adheres to the principles set out in the WMA Declaration of Helsinki. The protocol used to transform B cells from these anonymous donors with EBV was approved by IDIBELL's Committee of Biosecurity (CBS) on 5 May 2011 and the Ethics Committee of the University Hospital of Bellvitge (CEIC) on 28 May 2011.

The study population consisted of a retrospective series of *de novo* cases of DLBCL obtained from various centers in Spain. The study was reviewed and approved as being of minimal or no risk or as being exempt by each of the participating institutional review boards, and the overall collaborative study was approved by the institutional review board of the Spanish National Cancer Research Centre (CNIO), Madrid, Spain. The study protocol and sampling methods were approved by the Instituto de Salud Carlos III institutional review board in de-identified anonymous format. All cases positively stained for CD20. Cases diagnosed as T cell histiocyte-rich B cell lymphoma, primary mediastinal B cell lymphoma cases, cutaneous LBCL, intravascular LBCL and those histologically associated with a follicular lymphoma component were excluded.

### Cells

Viable peripheral blood mononuclear cells were isolated by Lymphoprep density gradient centrifugation from buffy coats from anonymous blood donors. Resting B cells were isolated by positive selection using CD19 MicroBeads (Miltenyi Biotec), or by depletion using a B Cell Isolation Kit (Miltenyi Biotec). For EBV-mediated transformation, isolated B cells were immortalized with the supernatant of the marmoset cell line B95.8 and with the EBV variant 2089 made from 293 cells carrying a recombinant B95.8 EBV genome (16). Preparations of the 2089 recombinant wild-type EBV with a GFP (green fluorescent protein) vinsert or viruses deleted for LMP-1 and EBNA-2 (17) were made from 293 cells carrying the recombinant B95.8 EBV genomes, and transfected with 0.5 μg BZLF1 (p509) + 0.5 μg gp110 (pRA). For B cell activation, isolated B cells were cultured 5 × 10^6^/3 ml per well of a 6-well plate with 50 ng/ml CD40L (Enzo Life Sciences) and 50 ng/ml IL-4 (Gentaur) and the B cell blasts were split weekly in a 1:2 ratio. The percentages of activated and proliferating B-cells were detected by CD86 expression measured by flow cytometry and tritiated thymidine incorporation, respectively.

### miRNA profiling and individual assays

For the miRNA expression analysis, cDNA synthesis and real-time quantitative polymerase chain reaction (qPCR) were performed using the miRCURY LNA Universal RT microRNA PCR system (Exiqon, Denmark) according to the manufacturer's instructions. miRNAs were screened using Ready-to-Use microRNA PCR Human Panel I V2.R from Exiqon. For each reverse transcriptase-PCR (RT-PCR) reaction, 30 ng of total RNA were used. Samples from RBLs and LCLs were analyzed in triplicates on a Roche LightCycler 480 real-time PCR system. Results were converted to relative values using the inter-plate calibrators included in the panels (log 2 ratios). Samples with Cp values equal or higher than 37 were considered as having the same value (i.e. 37 Cp), considering that above that threshold the amount of a particular miRNA is negligible. RBL and LCL average expression values were normalized with respect to the reference miRNA miR-103. Differentially expressed miRNAs (log FC > 2 or log FC < −2) were selected. Individual assays were also performed using probes from Exiqon. Nucleolar RNAs RNU44 and RNU48 were used for frozen samples assays.

### Quantitative RT-PCR (qRT-PCR)

For qRT-PCR of cellular genes, cDNA was produced with the SuperScript II Reverse Transcriptase (Invitrogen Co). Quantitative real-time PCR was done on a LightCycler 480 II System using LightCycler 480 SYBR Green Mix (Roche). Reactions were carried out in triplicate and qRT-PCR data were analyzed using the standard curve method. We used the housekeeping gene RPL38 and HPRT1 as a control. All primer sequences are listed in Supplementary Table S1.

### Chromatin immunoprecipitation (ChIP) assays

To test the binding of p65 NF-kB to miRNA promoters, as well as changes in histone H3K4me3 and histone H3K27me3, we performed ChIP assays as previously described (18). We used a rabbit polyclonal against the C-t of NF-kB p65 (sc-372, Santa Cruz Biotechnology), anti-histone H3K4me3 (17–614, Millipore) and anti-histone H3K27me3 (07–449, Millipore). Immunoprecipitated material was used for analyses of specific sequences by qRT-PCR (see primers sequences in Supplementary Table S1).

### DNA methylation analysis

Bisulfite pyrosequencing (BPS) was performed according to standard protocols and evaluated with the Pyro Q-CpG 1.0.9 program (Biotage, Uppsala, Sweden). Primer sequences for BPS PCR reactions are shown in Supplementary Table S1.

### ChIP-seq analysis

#### ChIP-seq sources and data processing

Sources of ChIP-seq data are detailed in Supplementary Table S2. We downloaded either aligned BED or BAM format files from public databases. BAM files were converted to BED format using BETools (bamToBed function) (19). When there was more than one replicate, they were concatenated to obtain wider coverage and greater sequence depth.

#### Analysis of differential association of histone modifications

Differential association (increased or decreased genomic location) of H3K4me3, H3K27me3 and H3K9me3 was analyzed using the MACS program (version 2.0.9; macs2diff function) (20) using the parameter settings: -g hs -nomodel -shiftsize = 75 -bdg -a 4 -q 0.005. Therefore, the *q*-value cutoff was established as 0.005. MACS’ macs2diff function establishes differential regions by comparing two treatment files corresponding to two conditions (in this case, lymphoblastoid cells and resting B cells), and comparing each of them against corresponding control ‘Input’ files. This comparison generates an output containing a list of ‘differentially bound locations’ with a ‘differential score’ (−log_10_ *q*-value), where a positive number means that the signal in lymphoblastoid cells is higher than in resting B cells, and a negative number means the opposite. Higher scores indicate a greater difference between the two cell types. Unique and consistently found differentially bound locations were considered for further analysis, e.g. correlation with expression. However, differential locations with a score (−log_10_ *q*-value) of exactly zero were excluded from the analysis. When more than one replicate of the aligned file was present, they were merged to ensure wider coverage and greater sequencing depth. Significantly differentially bound locations were annotated to the closest EnsEMBL (version-65) transcripts and genes (21) using BEDTools (closestBed function) (19). We calculated the Pearson's correlation coefficient between the differentially bound location scores, in which negative and positive values respectively indicate decreased and increased location scores in lymphoblastoid cells/resting B cells comparisons. Normalized read density wig files were produced using JavaGenomics Toolkit (http://palpant.us/java-genomics-toolkit/) and were used in UCSC (University of California, Santa Cruz) genome browser (http://genome.ucsc.edu/) to visualize read-density peaks around miRNA TSS.

### Transfection with miRNA mimics and siRNAs

A total of 5 nM miRNA mimics (Ambion) were transfected into LCLs using Lipofectamine RNAiMAX reagent (Invitrogen) following the manufacturer's protocol. Experiments were also performed using 5 nM control mimic. To silence p65, we used Silencer Select Pre-Designed siRNA (Life Technologies) against human RELA (p65), targeting exon 11 (ref. s11916) in parallel with a Silencer Select negative control in purified CD19+ cells, followed by addition of the EBV-containing supernatant 4 h after the cells were transfected with the siRNA. We used Lipofectamine RNAiMAX Transfection Reagent (Invitrogen) for efficient siRNA transfection. mRNA and protein levels were examined by qRT-PCR and western blot 2 and 3 days after siRNA transfection.

### Cell proliferation and apoptosis assays

To test the effects of transfection of specific mimics on DNA synthesis, we measured incorporation of 5′-bromo-2′-deoxyuridine (BrdU) 48 h after miRNA transfection. Cells were incubated in 15 μM BrdU during 30 min. Cells were fixed with 70% ethanol 1 h and permeabilized (phosphate buffered saline (PBS)-bovine serum albumin-Triton X-100 0.8% (PBT), 10 min, RT) and treated with 2 M HCl for 30 min, After DNA opening, HCl was neutralized by two 5-min washed with NaBo (0.1 M, pH 8.5) and two 5-min washes with PBT. Cells were incubated with anti-BrdU antibody (18 h at 4C, 1:1000 dilution) and anti-mouse Alexa-488 conjugated antibody was added to visualize the BrdU-positive nuclei. Cells were analyzed using fluorescence-activated cell sorting (FACS) (Beckman Coulter) and FlowJo software (Tree Star, Inc.). We also measured [3H]-thymidine incorporation, 48 h after miRNA transfection, cells were pulsed with 1 ml [3H]-thymidine (0.4 mCi/ml) for 4 h at 37C°. After washing three times with PBS, cells were incubated with 1 ml ice-cold 5% trichloroacetic acid (TCA) for 15 min at 4C°, washed three times with absolute methanol, air dried and the TCA-precipitable fraction was solubilized in 500 ml of 0.1 M NaOH-1% sodium dodecyl sulphate. [3H]-thymidine incorporation was determined with a scintillation counter. We used tetramethylrhodamine methyl ester (TMRM) to measure apoptosis. Forty eight hours after transfection with mimics, cells were incubated during 4 h with etoposide. Cells were washed two times with PBS and incubated 30 min at 37C° with TMRM. Cells were analyzed using FACS (Beckman Coulter) and FlowJo software (Tree Star, Inc.).

### Differential analysis from expression beadchips

Expression data were downloaded from NCBI (National Center for Biotechnology Information) GEO database (22) with accession number GSE30196 (23). Authors investigated global gene expression profiles in peripheral blood; hence, in this paper we focused on the samples of LCLs, and CD19-specific B cell subsets. Data analysis was performed on statistical environment R (24), using Bioconductor Package Genefilter with the purpose of applying non-specific filtering by IQR (interquartile range). Then first, processed data was utilized directly, the threshold for IQR was set to 0.5 and a Welch's *t*-test was carried out. Subsequently, data were selected taking a cut-off of *P*-value below 0.01, FDR (False Discovery Rate) below 0.05 and establishing fold-change value above 1.4 for overexpressed data and below 1/1.4 for downregulated genes.

### miRNA target prediction

To predict the potential targets of the dysregulated miRNAs, we used the algorithms available in miRWalk database (25), including information produced by eight established miRNA prediction programs on 3′ UTRs of all known genes of Human: RNA22, miRanda, miRDB, TargetScan, RNAhybrid, PITA, PICTAR and Diana-microT. Only those targets predicted by at least three databases were considered for further analysis.

### Differential expression analysis of microRNA microarrays

Microarray Expression data are Agilent Human MicroRNA microarray and come from a study by Martin-Pérez *et al.* (26) and data for lymph nodes (LN) (GSE23026) from the same research team. Data were processed and analyzed in R environment (24), using package AgimicroRNA (27) for processing microarrays and limma (28) for differential analysis. Thus, the workflow would be: (i) Preprocessing microarray using background method ‘half’, which establishes a value for negative data. (ii) Quantile normalization (iii) and default filtering method removing control and non-quality probes. (iv) An experimental Bayes moderated *t*-statistics test was carried out from limma package in order to observe differential expression in such data.

### Putative binding of NF-kB motifs

Possible occurrence of NF-kB subunit binding motifs in the region comprising 1000 bp upstream and 1000 bp downstream with respect to TSS was inspected using TRANSFAC matrices. The matches of the sequences against the set of TRANSFAC matrices were performed using R environment, specifically the functions countsPWM and matchPWM contained in the Bioconductor package Biostrings.

### Graphics and heatmaps

All graphs were created using Prism5 Graphpad. Heatmaps were generated from the expression data using the Genesis program from Graz University of Technology.

## RESULTS

### Screening of miRNAs during EBV-mediated conversion of RBLs to LCLs

**Figure 1.**
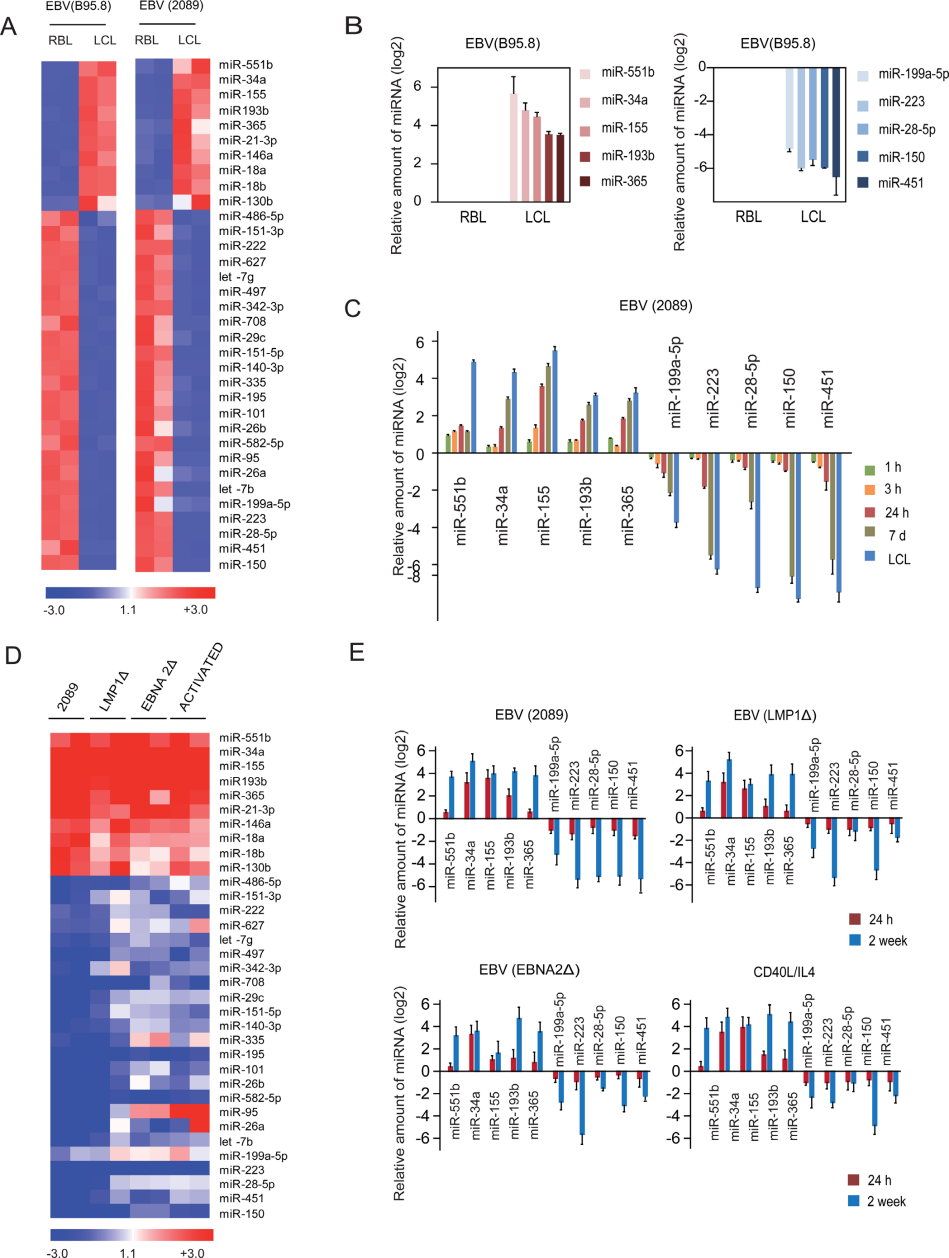
MicroRNA expression profiling during RBL-to-LCL transformation. (A) Heatmap including the data for the two RBL/LCL pairs of samples showing differential expression of miRNAs for each of the EBV systems (B95.8 EBV strain, left, and 2089 EBV strain, right). Only those miRNAs with an FC > 2 or FC < 0.5 were selected. Similar patterns of miRNA expression changes were obtained in the two situations. (B) Individual analysis of the top five upregulated and downregulated miRNAs upon B95.8 EBV infection using LNA probes and qRT-PCR. (C) Time-course analysis of the miRNA expression changes in the top upregulated and downregulated miRNAs using LNA probes and qRT-PCR. In this case we used the 2089 EBV strain, which has a very high yield of infection. (D) Heatmap showing differential expression of miRNAs between B cells after four different treatments (infected with wild-type 2089 EBV, EBV deficient for LMP-1, EBV deficient for EBNA-2 and stimulated with IL-4 /CD40L) with respect to resting B cells. The data represented in the heatmap correspond to the average of the fold change for each case. Similar patterns of miRNA expression were found in all samples. (E) Individual analysis of previously selected miRNAs using LNA probes and qRT-PCR for all four situations, as in the previous section.

To investigate the participation and role of miRNAs in EBV-mediated transformation of RBLs we first performed miRNA profiling with a qPCR-based panel containing over 375 miRNAs. The analysis included CD19+ samples before and after EBV infection, once they had become LCLs. For this analysis, we performed two independent sets of experiments with two forms of the EBV, the B95.8 strain and the 2089 form of EBV that infects B cells very efficiently (16), performing both in duplicate. Analysis of the results showed significant changes in expression of 34 miRNAs (Figure 1A and Supplementary Table S3). Remarkably, we observed predominant downregulation of miRNA expression. Specifically, 24 miRNAs displayed significant downregulation (including miR150, miR199a-5p, miR-223, miR-28-5p, miR451), whereas only 10 miRNAs became upregulated (including miR-551b, miR-34a, miR-155, miR-193b, miR-365).

Several of the upregulated and downregulated miRNAs have been previously described as being involved in the transformation of B cells or in lymphomagenesis. For instance, miR-155 is overexpressed during the acquisition of EBV-mediated latency III (29,30), and selective inhibition of miR-155 function specifically inhibits the growth of LCLs and DLBCL (31). Also, miR-34a has been shown to be strongly induced by EBV infection and expressed in many EBV and Kaposi's sarcoma-associated herpesvirus-infected lymphoma cell lines (10) and its inhibition impairs the growth of EBV-transformed cells. Of the downregulated miRNAs we identified miR-150, previously described as displaying an extremely low level of expression in Burkitt lymphoma cells and as inducing differentiation when ectopically expressed in Burkitt lymphoma lines (32).

We then performed qRT-PCR with specific LNA probes to test individual expression changes of those miRNAs displaying the largest changes between RBL and LCL, considering their potential relevance in the transformation process based on previous data. Comparing the levels of these miRNAs between RBLs and LCLs confirmed the results obtained in the high-throughput screening (Figure 1B). To determine the dynamics of changes in expression of these miRNAs we used the 2089 EBV strain (16), given that this strain yields a high level of infection and over 90% of B cells are positive for EBV transcription factor EBNA-2 a few hours after being exposed to the virus. Time-course analysis of expression showed that several of the miRNAs displayed changes even 24 h post-infection (for instance, miR-155 and miR-34a), before proliferation had started (Figure 1C), indicating that at least changes in these miRNAs might be related to the activation process or initial transformation steps.

To test the specificity of the changes in miRNA expression associated with EBV-mediated proliferation as well as the potential role of EBV proteins we screened in three additional conditions. First, we screened for B cells stimulated with IL-4 and CD40L, given that EBV-mediated transformation and B cell activation share common pathways (33). We found similar levels of cell activation, measured by cytometry analysis of the CD86 surface marker in IL-4/CD40L-stimulated cells and EBV-infected B cells (results not shown, but similar to those previously shown, see (9)). In parallel, we stimulated B cells with two recombinant forms of EBV in which the LMP-1 and EBNA-2 genes, the two best characterized EBV-encoded proteins, are deleted. Under these conditions, cells undergo a few divisions but do not acquire the capability of unlimited growth. Comparison of the miRNA expression levels of these three sample sets yielded similar profiles to those obtained with wild-type EBV (Figure 1D and Supplementary Table S4). In all cases, we observed a very similar profile for the set of upregulated miRNAs. In the case of downregulated miRNAs, we observed more variability, but in general the majority of miRNAs displayed changes in the same direction than the ones observed for wild-type EBV. These results suggest that all four conditions share a common pathway. It is well known that NF-kB is a common pathway for both IL-4/CD40L-stimulation and EBV infection, making it a strong candidate, even as a direct transcriptional regulator for some of them, to be that responsible for the changes observed in the panel of miRNAs. In fact, some of the misregulated miRNAs, like miR-155 (34), have previously been associated with the NF-kB pathway. It is also known that EBNA-1- and LMP-1-deficient EBV can still make use of the NF-kB pathway through LMP-2A (6). We also performed individual qRT-PCR analysis of the top upregulated and downregulated miRNAs at 24 h and 7 days post-infection of samples generated with wild-type EBV, EBNA-2- and LMP-1-deficient viral particles and samples generated through IL-4/CD40L activation. This enabled us to confirm that all four conditions have very similar effects on the changes that these miRNAs underwent (Figure 1E).

### Transcriptional and epigenetic control of miRNAs associated with EBV-mediated transformation of B cells

**Figure 2.**
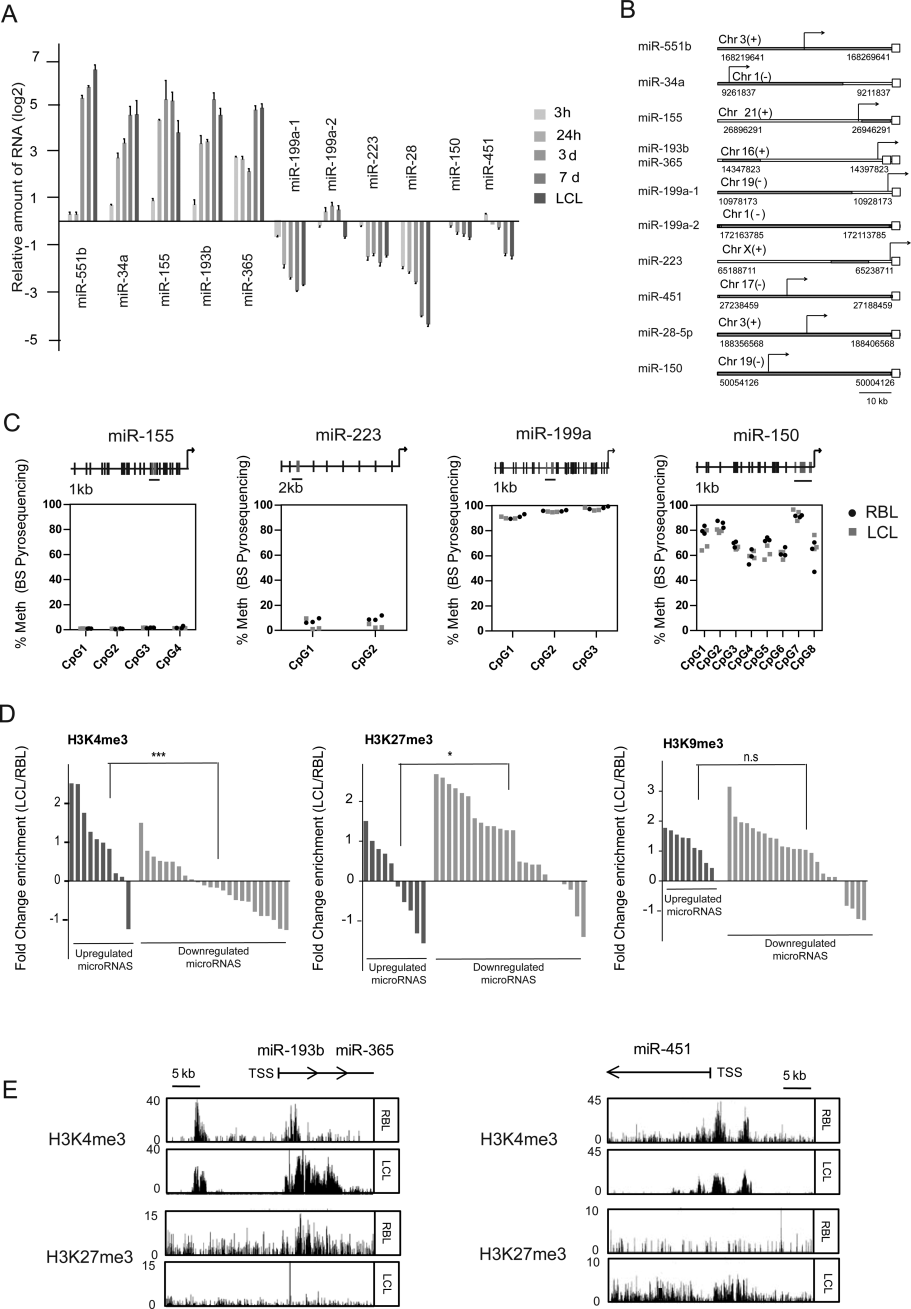
Transcriptional and epigenetic control of miRNAs in EBV-mediated transformation of RBLs. (A) qRT-PCR analysis of miRNA precursors (primary miRNAs) for the top five upregulated and downregulated miRNAs at different times (3 h, 1, 3, 7 days and LCLs) following infection of RBLs with EBV (2089 EBV strain). (B) Scheme depicting the genomic localization of selected miRNAs. Each empty square represents the mature form of a given miRNA. TSSs are indicated with an arrow. TSS locations were predicted using miRStart (http://mirstart.mbc.nctu.edu.tw/) (25). (C) Analysis of DNA methylation changes in CpGs by pyrosequencing. For each miRNA 2–8 consecutive CpGs were analyzed in RBLs and LCLs. The location of the analyzed CpGs is represented on top in relation with the TSS (indicated by an arrow). (D) Comparison of the ChIP-seq profiles for H3K4me3, H3K27me3 and H3K9me3 between RBLs and LCLs for all miRNAs of our study. A window of 5000 bp was inspected. The values correspond to the ratio between LCL and RBL of the average value using 50 bp segments within the 5000 bp window of each miRNA. Log2 values are represented. (E) Examples of the comparison of the ChIP-Seq profiles of two histone modifications (H3K4me3, active transcription; H3K27me3, repression) for one upregulated miRNA (miR-193b/miR-365) and one downregulated miRNA (miR-451) in RBLs and LCLs.

To determine whether miRNA expression changes observed during RBL to LCL conversion are a consequence of transcriptional regulation or due to modulation of miRNA stability, we determined by qRT-PCR the levels of long miRNA precursors, termed primary miRNAs (pri-miRNAs), during this process. The analysis of these precursors revealed similar dynamics in the changes to mature miRNAs levels, and most of the pri-miRNAs showed increased or decreased levels even before 24 h, indicating that these miRNAs are subject to transcriptional activation control (or repression). In the case of miR-199a we checked the pri-miRNAs corresponding to its two genomic locations, and only miR-199a1 (located in chromosome 19) displayed the expected changes in expression, discarding the other one as responsible for the observed changes in the mature form of this miRNA (Figure 2A).

The existence of transcriptional control in mediating the observed changes in miRNA expression suggests the potential participation of epigenetic mechanisms, together with changes in association of transcription factors. Previous results from our team had demonstrated the existence of DNA demethylation events in association with EBV-mediated transformation (8) as well as changes in histone modifications like H3K27me3 and H3K9me3 (9). We therefore tested the epigenetic profile around the TSS of several upregulated and downregulated miRNAs. To this end we first determined the TSS of all miRNAs displaying expression changes using miRStart (Figure 2B) (25). We then investigated the possible existence of DNA methylation changes near the TSS using BPS. For instance, for miR-155, one of the upregulated miRNAs, we found no detectable levels of methylation in the four CpG sites near its TSS in either RBLs or LCLs (Figure 2C). Other examples of miRNAs tested for methylation near the corresponding TSS included downregulated miRNAs like miR-223, miR-199a and miR-150, in which we observed variable levels of DNA methylation but no differences were found between RBL and LCL (Figure 2C). In summary, we did not observe any changes in DNA methylation at the TSS associated with miRNAs.

We also tested the levels of histone modifications like histone H3K4me3, H3K27me3 and H3K9me3, which are associated with transcriptional activation and repression, respectively. To this end, we compared ChIP-seq data between RBLs and LCLs (GSE19465 for RBLs, ENCODE data for GM12878 for LCLs, described in detail in the Materials and Methods). We noted changes in these marks that were compatible with those observed in the expression of these miRNAs. A general analysis across all up- and downregulated miRNAs revealed that both H3K4me3 and H3K27me3 show significant changes that are consistent with the change in expression (Figure 2D and Supplementary Table S5). Specifically, upregulated miRNAs generally displayed an increase in histone H3K4me3 and a decrease in histone H3K27me3, whereas downregulated miRNAs exhibited higher levels of histone H3K27me3 and lower levels of histone H3K4me3 (Figure 2D). In contrast, changes in H3K9me3 are less associated with the miRNA expression changes. For instance, we observed that miRNAs like miR-193b/miR-365, which become upregulated during RBL-to-LCL transformation, display an increase in H3K4me3 and a decrease in H3K27me3 (Figure 2E). For downregulated miRNAs, such as miR-223, miR-150 and miR-451 (Figure 2E and Supplementary Figure S1), the decrease in H3K4me3 was more evident than the increase in H3K27me3. In summary, our findings reinforce the notion of the existence of mechanisms that control gene transcription, which might complement the effects of direct control by transcription factors, including those involved in the NF-kB pathway.

### NF-kB is directly involved in regulation of both upregulation and downregulation of miRNAs in RBL-to-LCL transformation

**Figure 3.**
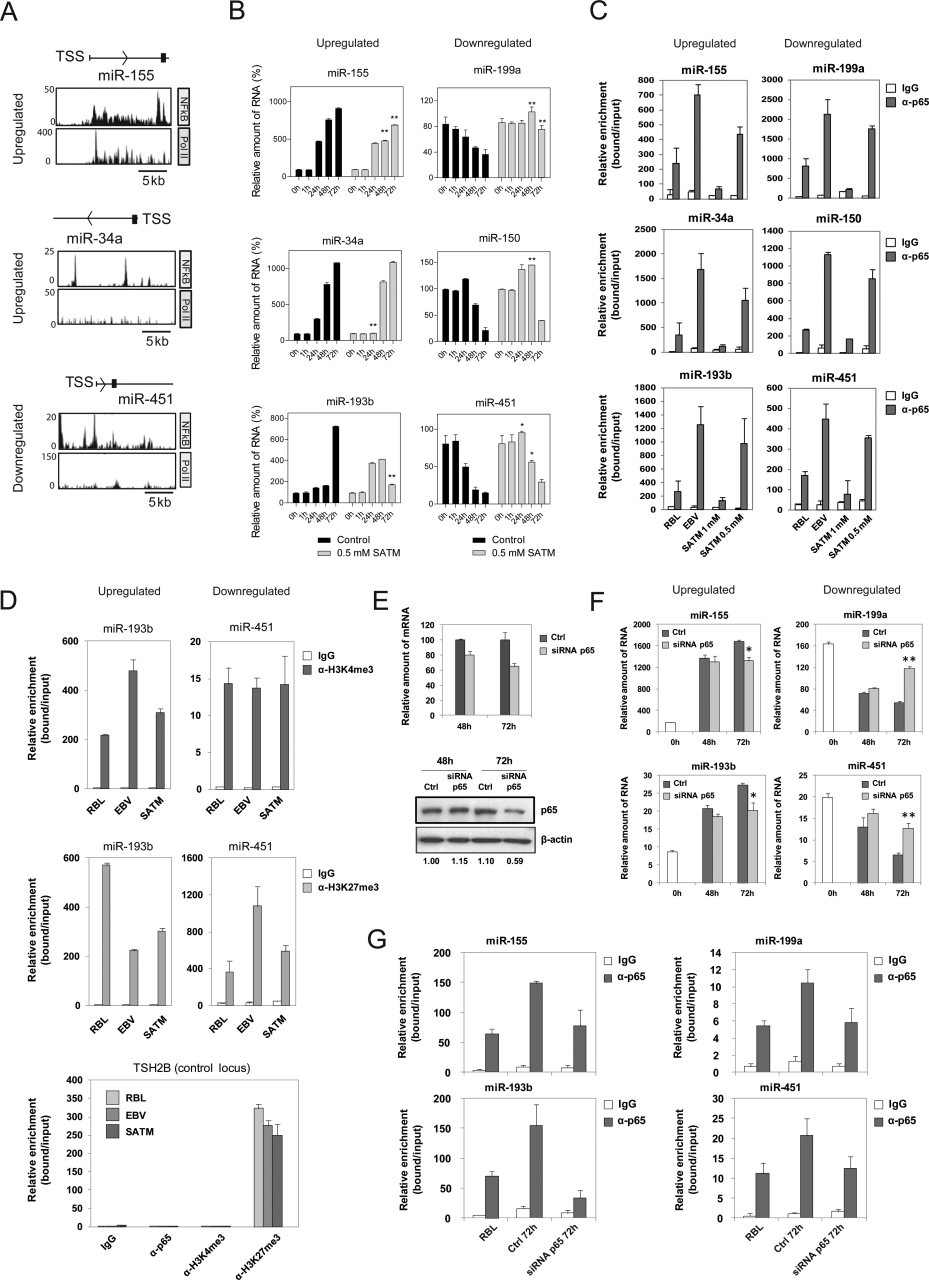
NF-kB dependence of miRNA expression changes. (A) ChIP-Seq analysis for NF-kB p65 and pol II in LCLs around the TSS of selected miRNAs, one upregulated (miR-155) and two downregulated (miR-34a and miR-451). (B) Effects of one of the NF-κB pathway inhibitors, SATM (at 500 μM), in the levels of selected upregulated and downregulated miRNAs in a time-course analysis of B cells infected with EBV. (C) ChIP assays for the above miRNAs showing the binding of the NF-kB p65 and pol II in B cells infected with EBV after 3 days and once they have become LCLs, as well as the effect following treatment with NF-kB pathway inhibitor SATM at two concentrations (500 μM and 1 mM). (D) ChIP assays demonstrating changes in histone H3K4me3 and histone H3K27me3 at the TSS of upregulated and downregulated miRNAs, as well as the effect following treatment with 1 mM SATM. (E) Effects of siRNA on p65 levels in B lymphocytes following transfection and EBV infection after 2 and 3 days, as analyzed by qRT-PCR (relative to the combined levels of the RPL38 and HPRT1 genes) and western blot (β-actin as loading control). Control samples correspond to B lymphocytes transfected with a control siRNA and were also infected with EBV. (F) Effect of p65 depletion on the expression changes of selected upregulated and downregulated miRNAs in a time-course analysis of B cells infected with EBV. (G) Effect of p65 depletion on their recruitment near the TSS of miRNAs, as demonstrated by ChIP assays.

To explore the potential involvement of the NF-kB pathway in the observed changes in miRNAs during the transformation of B cells we did several analyses. First, we investigated the enrichment of the consensus sites for NF-kB subunits (p65, p50, c-rel, etc.) in a window of 1000 bp upstream and downstream with respect to the TSS of miRNAs (determined from the miRStart database) (25). This showed these consensus binding sites to be present in the majority of miRNA TSSs (Supplementary Figure S2). We then determined the presence of peaks of p65 subunit of NF-kB and RNA pol II binding from ChIP-seq experiments in the proximity of the TSSs of all miRNAs using public data sets (see Supplementary Table S2). This revealed the presence of p65 peaks in 7 out of 10 upregulated miRNAs and 11 out of 24 downregulated miRNAs, indicating that p65 NF-kB is physically associated with the TSS of both upregulated and downregulated miRNAs and, therefore, perhaps involved both in activation and repression of miRNAs (Figure 3A and Supplementary Figure S3). To further explore the involvement of the NF-kB pathway in the activation and repression of these miRNAs, we treated RBLs with two different NF-κB inhibitors: Bay 11–7082 and sodium aurothiomalate (SATM) and investigated the effects on the expression changes on the aforementioned miRNAs. Bay 11–7082 has been commonly used as a specific NF-kB inhibitor, although recent data have questioned its specificity (35,36). SATM forms gold adducts with the cysteine residues of IKK (37) although its use as a NF-kB inhibitor has been more restricted. We first set up the concentration for each inhibitor by using LCLs and a wide range of concentrations. We selected 10 μM for Bay 11–7082 and 500 μM for SATM. Both compounds were able to impair changes in expression of upregulated and downregulated miRNAs, although Bay 11–7082 has a higher effect (Supplementary Figure S4 and Figure 3B), reinforcing the notion of the role of this pathway in mediating misregulation of these miRNAs.

We then confirmed the presence of p65 NF-kB at the TSS of these miRNAs, by performing ChIP assays with anti-p65 antibodies in CD19+ cells before and 72 h after infection with EBV and investigated the association of p65 at the genomic sequence around the TSS of both miRNAs that become upregulated (miR-155, miR-34a and miR-193b) (34) and downregulated (miR-199a, miR-150 and miR-451), focusing on sequences that display binding motifs for NF-kB. We also used the *TSH2B* as negative control. Interestingly, we observed specific enrichment of p65 at 72 h after EBV infection in both upregulated and downregulated miRNAs genes (Figure 3C), demonstrating the EBV-dependent association of p65 to both sets of miRNAs. Interestingly, binding of p65 was abrogated upon treatment with the NF-kB pathway inhibitor SATM (Figure 3C). For this and the following experiments we used SATM instead of Bay 11–7082, because the latter induces cell death, as also reported by others (38).

Finally, to test the implication of NF-kB in the histone modification-mediated regulation of these miRNAs we performed ChIP assays with the two histone modifications which had displayed significant changes in relation with their increase or decrease at the TSS of these miRNAs, i.e. histone H3K4me3 and histone H3K27me3. ChIP assays confirmed the decrease of histone H3K27me3 in miRNAs that become upregulated and their increase in miRNAs that are downregulated (Figure 3D, top). In contrast, we observed and increase of histone H3K4me3 in miRNAs that become upregulated, although changes in miRNAs that become downregulated were less strict (Figure 3D, bottom). These changes were partially or totally abrogated in the presence of NF-kB pathway inhibitor SATM (Figure 3D). These changes were not observed at control genes that are not targeted by p65 (Figure 3D).

Given the limited specificity of the two above inhibitors, as an unequivocal test of a potential causal relationship between NF-kB and miRNA expression changes in EBV-mediated B lymphocyte transformation, we investigated the consequences of ablating p65 expression in RBLs. To this end, we downregulated p65 levels in RBLs using transient transfection experiments with an siRNA that targets exon 11 of p65. In parallel, we used a control siRNA. Following transfection we infected both control siRNA- and p65 siRNA-treated cells with EBV. Under these conditions, we used qRT-PCR and western blot to check the effects on p65 levels 48 and 72 h after EBV infection. By this means, we were able to confirm that the level of p65 downregulation was close to 40% at 72 h at both mRNA and protein levels (Figure 3E). We examined the expression levels of these miRNAs following p65 depletion and found that both upregulation and downregulation of the miRNAs was partially impaired following siRNA-mediated p65 depletion (Figure 3F). We confirmed that siRNA-mediated downregulation of p65 resulted in decreased binding of p65 to the miRNAs-associated TSS (Figure 3G).

Together, our findings indicate that NF-kB is involved in both activation and repression of miRNAs, and that this process is mediated by changes in histone H3K4me3 and histone H3K27me3. Most of the reports have demonstrated a role for NF-kB as a transcriptional activator, although it has also been reported that it can act as a repressor (39).

### Analysis of miRNA expression in B cell lymphomas

**Figure 4.**
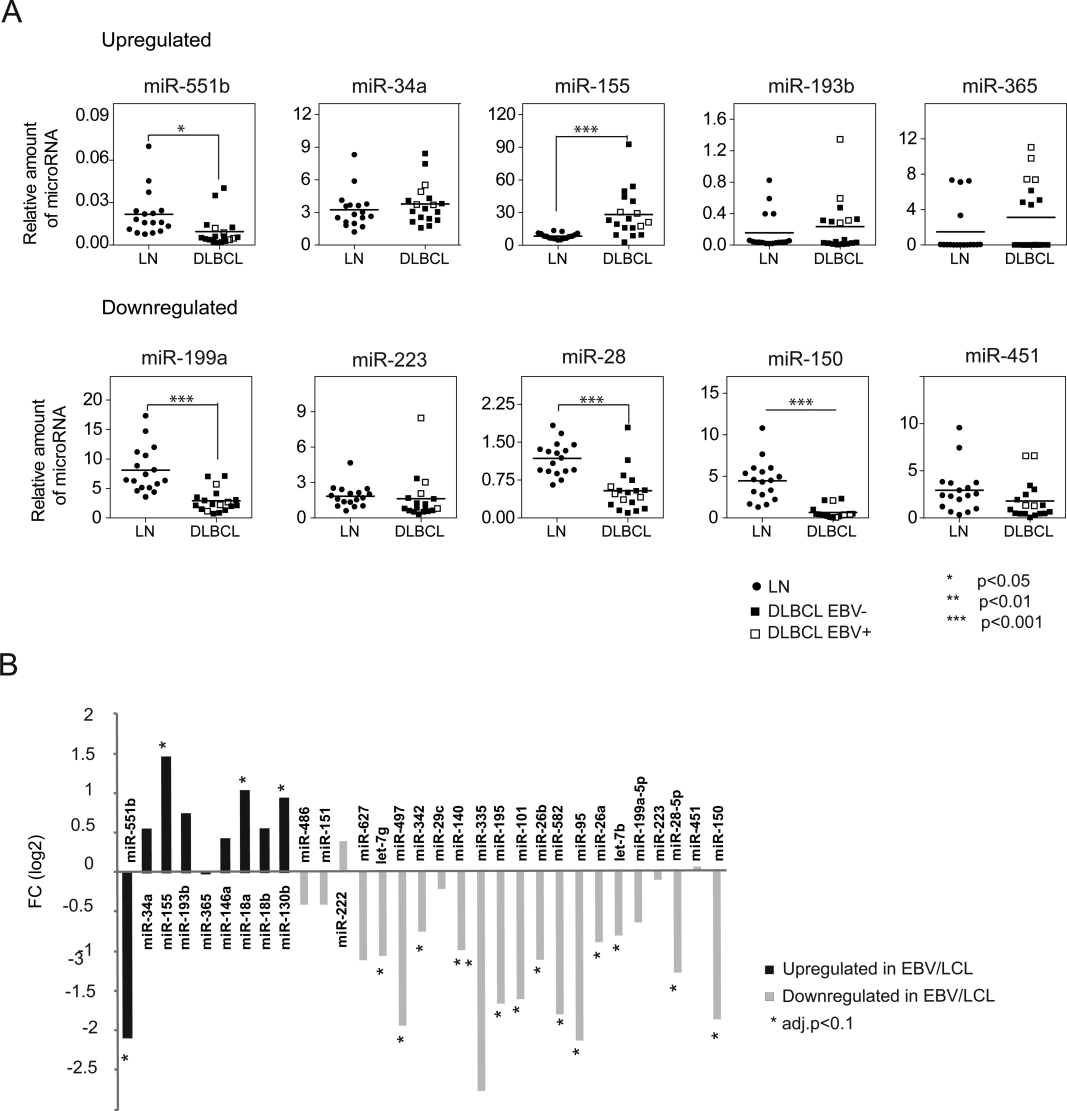
Comparison of the miRNA expression changes in primary DLBCLs. (A) Individual analysis of selected miRNAs in DLBCL and LN (negative control) cases using LNA probes and qRT-PCR. EBV positive and negative DLBCL cases are indicated. Fold changes were statistically significant for one upregulated miRNA (miR-155) and three downregulated miRNAs (miR-199a, miR-28 and miR-150) (***P < 0.0001). (B) Comparison of the miRNA expression data obtained by using a high-throughput analysis for miRNAs in a cohort of DLBCLs (26) and controls (LN) (GSE23026). Black and light gray bars correspond to upregulated and downregulated miRNAs respectively from the RBL versus LCL comparison.

As indicated, the pathogenic role of EBV and lymphomas is well established for Burkitt, Hodgkin and DLBCL, although the latter two have a less strict association than Burkitt lymphomas. On the other hand, of the lymphoid malignancies, those most clearly associated with NF-κB pathway are the ABC subgroup of DLBCL (40), Hodgkin lymphomas, primary mediastinal B cell lymphomas, gastric MALT lymphomas and multiple myeloma. Given these two facts, we decided to explore the relationship between the miRNAs whose expression changes in EBV-mediated transformation of RBLs and those obtained when comparing DLBCLs with normal LN, which are enriched in B cells. To this end, we first compared the expression status of the selection of the upregulated and downregulated miRNAs analyzed above and compared a cohort of 18 samples corresponding to DLBCL and 17 samples corresponding to control LN (Figure 4A). This comparison revealed that miR-155 is also upregulated in DLBCLs compared with LN and that miR-150, miR-199 and miR-28 are significantly downregulated in DLBCL with respect to LN. In parallel, we also used the miRNA expression data for DLBCL obtained by Martin-Pérez *et al.* (26); and LN (GSE23026) from the same research team and obtained the expression changes between these two groups. This comparison allowed us to confirm the data obtained by individual analysis of selected miRNA and identified additional miRNAs displaying a significant change in expression when comparing DLBCLs with LN similar to that observed during the EBV-mediated transformation of RBLs (Figure 4B and Supplementary Table S6). We also performed this analysis by separating the two DLBCL subgroups, ABC DLBCL and GC DLBCL. Interestingly, for the miRNAs that are misregulated in response to EBV-mediated transformation, we observed similar patterns of miRNA expression for both groups (Supplementary Figure S5).

### Functional effects of miRNAs in EBV-mediated transformation of B cells and in DLBCLs

**Figure 5.**
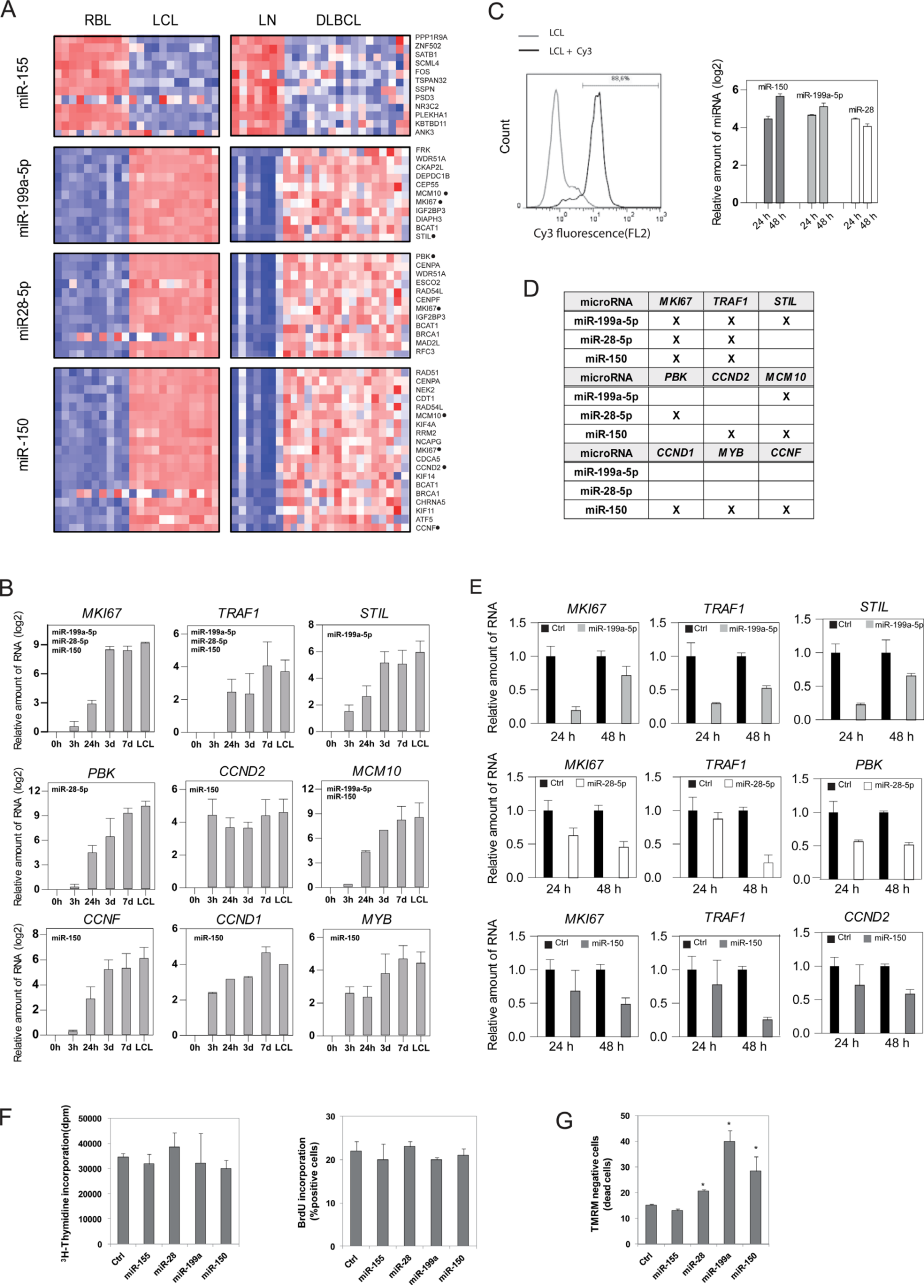
Expression changes of miRNA targets in EBV-mediated transformation of B cells and DLBCL. (A) Heatmap including expression data of putative targets in the RBL versus LCL and DLBCL versus LN comparisons. Represented targets include those predicted for at least 3 of 10 prediction algorithms (DIANA-mT, miRanda, miRDB, miRWalk, RNAhybrid, PCTAR4, PICTAR5, PITA, RNA22, TargetScan) in the miRWAlk program. Putative targets were compared with expression data from microarray data of RBL versus LCL (FC > 2, *P* < 0.05) and DLBCL versus LN (FC > 1.3, *P* < 0.05). Only targets common to both processes were represented. (B) Individual analysis for selected targets using qRT-PCR and a time-course series of B cells following EBV infection. (C) Efficiency of the experiments of miRNA mimic transfections. (D) Summary table indicating the miRNA/targets analyzed in functional experiments. (E) Effects of the introduction of mimics in LCLs for selected miRNAs (miR-150, miR-199a-5p and miR-28-5p) in the expression levels of their targets. (F) Analysis of tritiated thymidine and BrdU incorporation on MD209 cells following transfection with selected miRNA mimics (miR-155, miR-150, miR-199a-5p and miR-28-5p) and a control miRNA mimic (G) Analysis of apoptosis levels following transfection with the aforementioned selected miRNA mimics and measured by TMRM fluorescence.

Changes in the levels of miRNAs are likely to affect the levels of their targets. To identify miRNA targets, we retrieved a list of putative targets using miRWalk (41), which contains prediction databases like TargetScan (42), miRDB (43) and others, as well as information about validated targets. We then linked the list of potential targets with expression data from RBL versus LCL comparisons (GSE30916) (23) and with expression data corresponding to a cohort of 17 DLBCL samples and 7 control LN (44), assuming an inverse relationship between the levels of a given miRNA and the expression levels of its targets. To select targets we chose those predicted with a minimum of three hits in different databases from miRWalk and at least 2-fold change for overexpressed genes or 0.5-fold change for downregulated genes (Figure 5A and Supplementary Table S7). We concentrated our efforts on those miRNAs for which we had observed changes in the same direction when comparing RBL versus LCLs and DLBCL versus LN, i.e. miR-155, among the tested upregulated miRNAs, and downregulated miRNAs mir-199a-5p, miR-28-5p and miR-150. We observed a number of putative targets relevant to B cell transformation. For instance, *MKI67* and *TRAF1* are targeted by miR-150, miR-199a-5p and miR-28-5p. *MKI67* encodes for a protein that is considered a classical marker for cell proliferation (45) and TRAF1 is a key factor in EBV-mediated transformation (46) and in lymphomagenesis (47). Other examples include *MCM10* and *CCND1* also targeted by miR-199a-5p, *PBK*, that promotes tumour cell proliferation through p38 MAPK activity (48) that is targeted by miR-28-5p and cyclin genes *CCND1* and *CCND2* targeted by miR-150. We confirmed changes in expression of these targets over a time course in B cells following EBV infection (Figure 5B).

To confirm the direct effects of the aforementioned miRNAs on the levels of their putative targets, we focused on the downregulated miRNAs. We transfected mimics for miR-150, miR-199 and miR-28 in LCLs. The transfection efficiency of mature miRNAs was assessed by FACS analysis using control fluorophore-labeled miRNAs, which revealed that over 88% of LCLs became positive (Figure 5C). qRT-PCR of each miRNA showed peak high expression levels with respect to untransfected LCLs at 24 and 48 h (Figure 5C). We then investigated the effects on the mRNA levels of their targets. Several of these targets had significantly reduced mRNA levels following mimic transfection (Figure 5E), indicating a role of the changes in these miRNAs with respect to their levels.

Finally, we tested the effects of these miRNAs on cell proliferation and apoptosis. We first tested the impact of transfecting DLBCL cell line MD901 with mimics for miR-155 (upregulated during EBV-mediated B cell transformation), miR-150, miR-199 and miR-28 (downregulated) measuring incorporation of BrdU or tritiated thymidine. We were unable to observe any significant effect on cell proliferation (Figure 5F). In contrast, when we investigated the effects on induced apoptosis following transfection with these mimics, we observed that ectopic expression of miR-155 increased the resistance to apoptosis, whereas overexpression of miR-150, miR-199 and miR-28 increased the levels of apoptotic cells (Figure 5G), indicating that their downregulation following infection with EBV reduces the propensity to undergo apoptosis during EBV-mediated transformation of B cells.

## DISCUSSION

Our results provide evidence of the existence of widespread changes of miRNA expression in association with EBV-mediated transformation of RBLs. It is notable that downregulation of miRNAs is predominant with respect to miRNA overexpression. In addition, comparison of these results with those obtained by activating and inducing proliferation with the T cell-derived mitogens CD40L and IL-4, as well as with EBV particles deficient for EBNA-2 and LMP-1, suggests that the process is driven by a pathway that is common to all four situations, i.e. the stimulation of the NF-kB pathway. This hypothesis is reinforced by our findings on the presence of binding motifs of NF-kB subunits, NF-kB p65 ChIP-seq data near the TSS of miRNAs, ChIP assays on selected up- and downregulated miRNAs, as well as results about the inhibition of this pathway and the effects on miRNA levels and reduced p65 binding. Several of the miRNA targets are related to cell transformation or proliferation. In addition, the significant overlap between the miRNA expression changes in EBV-related B cell transformation and DLBCL versus LN reinforces the notion of the relevance of this pathway to B cell transformation and especially in lymphomagenesis.

Several studies have previously addressed the effects of EBV infection on the miRNAs of host B cells. Changes in various miRNAs have been described, including upregulation of miRNAs like miR-155 (29,30) and miR-34a (10), and downregulation of miRNAs like miR-150 (32). Our own data have shown changes in these miRNAs as well. The global analysis shows that miRNA downregulation is predominant with respect to miRNA upregulation. miRNAs have negative effects on gene expression, so an overall decrease in miRNA levels suggests that some of the constraints for gene repression are relaxed. This observation is compatible with the general notion that transformation of RBLs to LCLs results in overexpression of many genes. These data are also consistent with our own observations about epigenetic control when analyzing DNA methylation and histone modification changes during the EBV-mediated transformation of B cells. In the case of DNA methylation we found that hypomethylation, and not hypermethylation, occurs during this process (8) again reinforcing the idea of relaxation of gene control. For histone modifications and accessibility to endonucleases we also observed changes that are compatible with increased gene expression, specifically a decrease in heterochromatic histone modifications and increased accessibility (9).

Our findings also indicate that the changes observed for RBL transformed with wild-type EBV are common to other similar situations. We noted a similar pattern of miRNA misregulation in B cells stimulated with IL-4/CD40L and in B cells infected with EBV deficient for LMP-1 and EBNA-2, the best characterized EBV proteins. In the first case, cells are stimulated to proliferate at a similar rate to cells infected with EBV. In B cells infected with these defective EBV particles, the initial steps toward transformation take place but proliferation rates are much lower. However, all situations share the stimulation of the NF-kB pathway and the commonalities in the misregulation of miRNAs suggest the role of this pathway. Although our study has focused on the elements within the canonical pathway, EBV and CD40L also stimulated the non-canonical pathway (49,50), which may also be involved in miRNA misregulation. It is then likely that LMP-1-deficient EBV make use of the non-canonical NF-kB pathway and this could explain stimulation of common sets of miRNAs. This had been previously demonstrated for miR-155 (34), in which the two binding sites at the miR-155 promoter recruit NF-kB, which is stimulated by LMP-1. In the case of EBNA-2- and LMP-1-deficient EBV particles, it is thought that stimulation of the NF-kB pathway occurs through LMP2A (6). The direct role of NF-kB in the regulation of these miRNAs is supported by the results obtained with both ChIP-seq data and individual ChIP assays and the observation that inhibitors for the NF-kB pathway impair both miRNA expression changes, as well as the binding of p65 and the changes in histone modifications at the mRNA genomic sites during the transformation of RBL with EBV. The implication of the NF-kB in directly targeting miRNAs has previously reported (34), however, to the best of our knowledge, our study is the first evidence demonstrating a direct role of this pathway in both targeting up- and downregulated miRNAs, as well as the finding of direct changes in the histone modification marks around the TSS of both groups of miRNAs. Previous studies have shown the ability of EBV and LMP-2 to upregulate DNMT3b and drive hypermethylation in gastric cancer (51), however, during infection of B cells from peripheral blood, we have been able to identify demethylation events (8) and there are no changes at the miRNA genomic sites.

The NF-kB pathway is relevant in the context of EBV-transformation of B cells and is considered to be a major pathway in lymphomagenesis, particularly in certain types of lymphomas, including DLBCL, which are also known to be associated with EBV infection. Comparing the miRNA displaying significant change during the EBV-mediated transformation of RBLs and those from the DLBCL and LN analysis revealed striking resemblances, suggesting that the type of changes in miRNAs are common to the two processes. This also applies to large sets of the predicted targets for these miRNAs, reinforcing the notion of the relevance of EBV and NF-kB in the pathogenesis of DLBCL. Earlier studies have shown that ABC DLBCL are more dependent on the NF-kB pathway (40). In this sense, it was relevant to compare the miRNA expression profiles of ABC DLBCL versus those from GC DLBCL. However, we did not observe a significant difference between these two. Recent studies have demonstrated that mutations in NF-kB related genes account for deregulation of the NF-kB pathway also in GC DLBCL (52).

There is increasing evidence of a role for miRNAs in proper B cell differentiation and in relation with the pathogenesis of B cell lymphomas (13). Interestingly, among the misregulated miRNAs in our model, i.e. the EBV-mediated transformation of B cells, there are several well studied miRNAs known for their relevance in the biology and cell cycle within the B cell compartment. For instance, miR-150 is associated with B cell differentiation and its upregulation is negatively associated with the expression of C-MYB (53). MiR-34a, one of the top upregulated miRNAs in EBV-mediated B cell transformation, is known to target Foxp1 required for B cell differentiation (54). In this context, miR-155 has also been associated with B cell differentiation (55). Quiescent differentiated B cells have a restricted transcription program in which only a limited set of genes (ubiquitously expressed and cell type-specific genes) are expressed. This progressive restriction of the transcription program, is well known to occur during differentiation. EBV-mediated transformation could therefore be viewed as a process inverse to that occurring during differentiation, and some of the mechanisms involved are likely to act in an opposite direction to those driving differentiation. Therefore, the activation of the aforementioned miRNAs, and the downregulation of a large number of miRNAs leading to the loosening of repression of many genes would be consistent with the type of changes occurring in transformed B cells. Our analysis of miRNA targets has led to the identification of genes that may play a key role in the transformation process and in maintaining the phenotype in lymphoma cells, including *MKI67* and *TRAF*, targeted by at least three of the downregulated miRNAs, and others like *CCND1*, *STIL* and *PBK* targeted by miR-150, miR-199a-5p and miR-28, respectively. Overall, our findings indicate a close relationship in the miRNA-mediated acquisition of the phenotype in B cells and a connection between the transformation process and the phenotype in lymphomas.

## SUPPLEMENTARY DATA

Supplementary Data are available at NAR Online.

SUPPLEMENTARY DATA

## References

[1] Bornkamm G.W. (2009). Epstein-Barr virus and its role in the pathogenesis of Burkitt's lymphoma: an unresolved issue. Semin. Cancer Biol..

[2] Kaye K.M., Izumi K.M., Kieff E. (1993). Epstein-Barr virus latent membrane protein 1 is essential for B-lymphocyte growth transformation. Proc. Natl. Acad. Sci. U.S.A..

[3] Kilger E., Kieser A., Baumann M., Hammerschmidt W. (1998). Epstein-Barr virus-mediated B-cell proliferation is dependent upon latent membrane protein 1, which simulates an activated CD40 receptor. EMBO J..

[4] Feederle R., Haar J., Bernhardt K., Linnstaedt S.D., Bannert H., Lips H., Cullen B.R., Delecluse H.J. (2011). The members of an Epstein-Barr virus microRNA cluster cooperate to transform B lymphocytes. J. Virol..

[5] Hollyoake M., Stuhler A., Farrell P., Gordon J., Sinclair A. (1995). The normal cell cycle activation program is exploited during the infection of quiescent B lymphocytes by Epstein-Barr virus. Cancer Res..

[6] Stewart S., Dawson C.W., Takada K., Curnow J., Moody C.A., Sixbey J.W., Young L.S. (2004). Epstein-Barr virus-encoded LMP2A regulates viral and cellular gene expression by modulation of the NF-kappaB transcription factor pathway. Proc. Natl. Acad. Sci. U.S.A..

[7] Niller H.H., Wolf H., Minarovits J. (2009). Epigenetic dysregulation of the host cell genome in Epstein-Barr virus-associated neoplasia. Semin. Cancer Biol..

[8] Hernando H., Shannon-Lowe C., Islam A.B., Al-Shahrour F., Rodriguez-Ubreva J., Rodriguez-Cortez V.C., Javierre B.M., Mangas C., Fernandez A.F., Parra M. (2013). The B cell transcription program mediates hypomethylation and overexpression of key genes in Epstein-Barr virus-associated proliferative conversion. Genome Biol..

[9] Hernando H., Islam A.B., Rodriguez-Ubreva J., Forne I., Ciudad L., Imhof A., Shannon-Lowe C., Ballestar E. (2013). Epstein-Barr virus-mediated transformation of B cells induces global chromatin changes independent to the acquisition of proliferation. Nucleic Acids Res..

[10] Forte E., Salinas R.E., Chang C., Zhou T., Linnstaedt S.D., Gottwein E., Jacobs C., Jima D., Li Q.J., Dave S.S. (2012). The Epstein-Barr virus (EBV)-induced tumor suppressor microRNA MiR-34a is growth promoting in EBV-infected B cells. J. Virol..

[11] Eis P.S., Tam W., Sun L., Chadburn A., Li Z., Gomez M.F., Lund E., Dahlberg J.E. (2005). Accumulation of miR-155 and BIC RNA in human B cell lymphomas. Proc. Natl. Acad. Sci. U.S.A..

[12] Arribas A.J., Gomez-Abad C., Sanchez-Beato M., Martinez N., Dilisio L., Casado F., Cruz M.A., Algara P., Piris M.A., Mollejo M. (2013). Splenic marginal zone lymphoma: comprehensive analysis of gene expression and miRNA profiling. Mod. Pathol..

[13] Di Lisio L., Martinez N., Montes-Moreno S., Piris-Villaespesa M., Sanchez-Beato M., Piris M.A. (2012). The role of miRNAs in the pathogenesis and diagnosis of B-cell lymphomas. Blood.

[14] Campo E., Swerdlow S.H., Harris N.L., Pileri S., Stein H., Jaffe E.S. (2011). The 2008 WHO classification of lymphoid neoplasms and beyond: evolving concepts and practical applications. Blood.

[15] Rosenwald A., Wright G., Leroy K., Yu X., Gaulard P., Gascoyne R.D., Chan W.C., Zhao T., Haioun C., Greiner T.C. (2003). Molecular diagnosis of primary mediastinal B cell lymphoma identifies a clinically favorable subgroup of diffuse large B cell lymphoma related to Hodgkin lymphoma. J. Exp. Med..

[16] Delecluse H.J., Hilsendegen T., Pich D., Zeidler R., Hammerschmidt W. (1998). Propagation and recovery of intact, infectious Epstein-Barr virus from prokaryotic to human cells. Proc. Natl. Acad. Sci. U.S.A..

[17] Feederle R., Bartlett E.J., Delecluse H.J. (2010). Epstein-Barr virus genetics: talking about the BAC generation. Herpesviridae.

[18] Ballestar E., Paz M.F., Valle L., Wei S., Fraga M.F., Espada J., Cigudosa J.C., Huang T.H., Esteller M. (2003). Methyl-CpG binding proteins identify novel sites of epigenetic inactivation in human cancer. EMBO J..

[19] Quinlan A.R., Hall I.M. (2010). BEDTools: a flexible suite of utilities for comparing genomic features. Bioinformatics.

[20] Zhang Y., Liu T., Meyer C.A., Eeckhoute J., Johnson D.S., Bernstein B.E., Nusbaum C., Myers R.M., Brown M., Li W. (2008). Model-based analysis of ChIP-Seq (MACS). Genome Biol..

[21] Hubbard T.J., Aken B.L., Beal K., Ballester B., Caccamo M., Chen Y., Clarke L., Coates G., Cunningham F., Cutts T. (2007). Ensembl 2007. Nucleic Acids Res..

[22] Barrett T., Wilhite S.E., Ledoux P., Evangelista C., Kim I.F., Tomashevsky M., Marshall K.A., Phillippy K.H., Sherman P.M., Holko M. (2013). NCBI GEO: archive for functional genomics data sets–update. Nucleic Acids Res..

[23] Min J.L., Barrett A., Watts T., Pettersson F.H., Lockstone H.E., Lindgren C.M., Taylor J.M., Allen M., Zondervan K.T., McCarthy M.I. (2010). Variability of gene expression profiles in human blood and lymphoblastoid cell lines. BMC Genom..

[24] Dessau R.B., Pipper C.B. (2008). [‘‘R”–project for statistical computing]. Ugeskr Laeger.

[25] Chien C.H., Sun Y.M., Chang W.C., Chiang-Hsieh P.Y., Lee T.Y., Tsai W.C., Horng J.T., Tsou A.P., Huang H.D. (2011). Identifying transcriptional start sites of human microRNAs based on high-throughput sequencing data. Nucleic Acids Res..

[26] Martin-Perez D., Vargiu P., Montes-Moreno S., Leon E.A., Rodriguez-Pinilla S.M., Lisio L.D., Martinez N., Rodriguez R., Mollejo M., Castellvi J. (2012). Epstein-Barr virus microRNAs repress BCL6 expression in diffuse large B-cell lymphoma. Leukemia.

[27] Lopez-Romero P. (2011). Pre-processing and differential expression analysis of Agilent microRNA arrays using the AgiMicroRna Bioconductor library. BMC Genom..

[28] Lymma G.K., Gentleman R., Carey, V., Dudoit, S., Irizarry, R. and Huber, W. (eds.) (2005). Limma: linear models for microarray data. Bioinformatics and Computational Biology Solutions using R and Bioconductor.

[29] Yin Q., McBride J., Fewell C., Lacey M., Wang X., Lin Z., Cameron J., Flemington E.K. (2008). MicroRNA-155 is an Epstein-Barr virus-induced gene that modulates Epstein-Barr virus-regulated gene expression pathways. J. Virol..

[30] Jiang J., Lee E.J., Schmittgen T.D. (2006). Increased expression of microRNA-155 in Epstein-Barr virus transformed lymphoblastoid cell lines. Genes Chromosomes Cancer.

[31] Linnstaedt S.D., Gottwein E., Skalsky R.L., Luftig M.A., Cullen B.R. (2010). Virally induced cellular microRNA miR-155 plays a key role in B-cell immortalization by Epstein-Barr virus. J. Virol..

[32] Chen S., Wang Z., Dai X., Pan J., Ge J., Han X., Wu Z., Zhou X., Zhao T. (2013). Re-expression of microRNA-150 induces EBV-positive Burkitt lymphoma differentiation by modulating c-Myb in vitro. Cancer Sci..

[33] Thorley-Lawson D.A. (2001). Epstein-Barr virus: exploiting the immune system. Nat. Rev. Immunol..

[34] Gatto G., Rossi A., Rossi D., Kroening S., Bonatti S., Mallardo M. (2008). Epstein-Barr virus latent membrane protein 1 trans-activates miR-155 transcription through the NF-kappaB pathway. Nucleic Acids Res..

[35] Strickson S., Campbell D.G., Emmerich C.H., Knebel A., Plater L., Ritorto M.S., Shpiro N., Cohen P. (2013). The anti-inflammatory drug BAY 11–7082 suppresses the MyD88-dependent signalling network by targeting the ubiquitin system. Biochem. J..

[36] Krishnan N., Bencze G., Cohen P., Tonks N.K. (2013). The anti-inflammatory compound BAY-11–7082 is a potent inhibitor of protein tyrosine phosphatases. FEBS J..

[37] Jeon K.I., Jeong J.Y., Jue D.M. (2000). Thiol-reactive metal compounds inhibit NF-kappa B activation by blocking I kappa B kinase. J. Immunol..

[38] Rauert-Wunderlich H., Siegmund D., Maier E., Giner T., Bargou R.C., Wajant H., Stuhmer T. (2013). The IKK inhibitor Bay 11–7082 induces cell death independent from inhibition of activation of NFkappaB transcription factors. PLoS ONE.

[39] Mott J.L., Kurita S., Cazanave S.C., Bronk S.F., Werneburg N.W., Fernandez-Zapico M.E. (2010). Transcriptional suppression of mir-29b-1/mir-29a promoter by c-Myc, hedgehog, and NF-kappaB. J. Cell. Biochem..

[40] Davis R.E., Brown K.D., Siebenlist U., Staudt L.M. (2001). Constitutive nuclear factor kappaB activity is required for survival of activated B cell-like diffuse large B cell lymphoma cells. J. Exp. Med..

[41] Dweep H., Sticht C., Pandey P., Gretz N. (2011). miRWalk–database: prediction of possible miRNA binding sites by ‘walking’ the genes of three genomes. J. Biomed. Inform..

[42] Lewis B.P., Burge C.B., Bartel D.P. (2005). Conserved seed pairing, often flanked by adenosines, indicates that thousands of human genes are microRNA targets. Cell.

[43] Wang X. (2008). miRDB: a microRNA target prediction and functional annotation database with a wiki interface. RNA.

[44] Di Lisio L., Gomez-Lopez G., Sanchez-Beato M., Gomez-Abad C., Rodriguez M.E., Villuendas R., Ferreira B.I., Carro A., Rico D., Mollejo M. Mantle cell lymphoma: transcriptional regulation by microRNAs. Leukemia.

[45] Gerdes J. (1990). Ki-67 and other proliferation markers useful for immunohistological diagnostic and prognostic evaluations in human malignancies. Semin. Cancer Biol..

[46] Mosialos G., Birkenbach M., Yalamanchili R., VanArsdale T., Ware C., Kieff E. (1995). The Epstein-Barr virus transforming protein LMP1 engages signaling proteins for the tumor necrosis factor receptor family. Cell.

[47] Zhang B., Wang Z., Li T., Tsitsikov E.N., Ding H.F. (2007). NF-kappaB2 mutation targets TRAF1 to induce lymphomagenesis. Blood.

[48] Ayllon V., O'Connor R. (2007). PBK/TOPK promotes tumour cell proliferation through p38 MAPK activity and regulation of the DNA damage response. Oncogene.

[49] Homig-Holzel C., Hojer C., Rastelli J., Casola S., Strobl L.J., Muller W., Quintanilla-Martinez L., Gewies A., Ruland J., Rajewsky K. (2008). Constitutive CD40 signaling in B cells selectively activates the noncanonical NF-kappaB pathway and promotes lymphomagenesis. J. Exp. Med..

[50] Luftig M., Yasui T., Soni V., Kang M.S., Jacobson N., Cahir-McFarland E., Seed B., Kieff E. (2004). Epstein-Barr virus latent infection membrane protein 1 TRAF-binding site induces NIK/IKK alpha-dependent noncanonical NF-kappaB activation. Proc. Natl. Acad. Sci. U.S.A..

[51] Zhao J., Liang Q., Cheung K.F., Kang W., Lung R.W., Tong J.H., To K.F., Sung J.J., Yu J. Genome-wide identification of Epstein-Barr virus-driven promoter methylation profiles of human genes in gastric cancer cells. Cancer.

[52] Compagno M., Lim W.K., Grunn A., Nandula S.V., Brahmachary M., Shen Q., Bertoni F., Ponzoni M., Scandurra M., Califano A. (2009). Mutations of multiple genes cause deregulation of NF-kappaB in diffuse large B-cell lymphoma. Nature.

[53] Xiao C., Calado D.P., Galler G., Thai T.H., Patterson H.C., Wang J., Rajewsky N., Bender T.P., Rajewsky K. (2007). MiR-150 controls B cell differentiation by targeting the transcription factor c-Myb. Cell.

[54] Rao D.S., O'Connell R.M., Chaudhuri A.A., Garcia-Flores Y., Geiger T.L., Baltimore D. MicroRNA-34a perturbs B lymphocyte development by repressing the forkhead box transcription factor Foxp1. Immunity.

[55] Vigorito E., Perks K.L., Abreu-Goodger C., Bunting S., Xiang Z., Kohlhaas S., Das P.P., Miska E.A., Rodriguez A., Bradley A. (2007). microRNA-155 regulates the generation of immunoglobulin class-switched plasma cells. Immunity.

