# Supplementary material for: NF-κB directly mediates epigenetic deregulation of common microRNAs in Epstein-Barr virus-mediated transformation of B-cells and in lymphomas
Source: Nucleic Acids Res. 2014 Sep 8;42(17):11025–39. doi: 10.1093/nar/gku826 (PMC4176189; doi:10.1093/nar/gku826)
Supplement: SUPPLEMENTARY DATA [file supp_42_17_11025__index.html]

NF-κB directly mediates epigenetic deregulation of common microRNAs in Epstein-Barr virus-mediated transformation of B-cells and in lymphomas — NF-κB directly mediates epigenetic deregulation of common microRNAs in Epstein-Barr virus-mediated transformation of B-cells and in lymphomas — SUPPLEMENTARY DATA 

# NF-κB directly mediates epigenetic deregulation of common microRNAs in Epstein-Barr virus-mediated transformation of B-cells and in lymphomas

## SUPPLEMENTARY DATA

**Files in this Data Supplement:**

- SUPPLEMENTARY DATA
