# Supplementary material for: NF-κB directly mediates epigenetic deregulation of common microRNAs in Epstein-Barr virus-mediated transformation of B-cells and in lymphomas
Source: Nucleic Acids Res. 2014 Sep 8;42(17):11025–39. doi: 10.1093/nar/gku826 (PMC4176189; doi:10.1093/nar/gku826)

Legends to Supplementary Figures

Supplementary Figure 1. Examples of the comparison of the ChIP-Seq profiles of two histone modifications (H3K4me3, active transcription; H3K27me3, repression) for upregulated miRNAs (miR-551b, miR-34a, miR-155 and miR-193b/miR-365) and downregulated miRNAs (miR-199a1, miR-223, miR-28-5p, miR-150 and miR-451) in RBLs and LCLs. Data was obtained from public databases (GSE19465 for RBLs, ENCODE data for GM12878 for LCLs) and was normalized and processed as described in Material and Methods.

Supplementary Figure 2. Analysis of the presence of NF-kB subunit binding motifs (from TRANSFAC database) at a 1000 bp window around the estimated TSS of the miRNAs (A) Analysis of the presence of NF-kB subunit binding motifs (from TRANSFAC database) at a 1000 bp window around the estimated TSS of the miRNAs

Supplementary Figure 3. ChIP-Seq profiles for NF-kB p65 and pol II in LCLs around the TSS of selected miRNAs that become upregulated and downregulated in EBV-mediated transformation of B cells. Upregulated and downregulated miRNAs are labeled in red and blue respectively.

Supplementary Figure 4. Effects of one of the NF-κB pathway inhibitors, Bay 11-7082 (10 μM), in the levels of selected upregulated and downregulated miRNAs in a time-course analysis of B-cells infected with EBV.

Supplementary Figure 5. Comparison of the miRNA expression data obtained by using a high-throughput analysis for miRNAs (see reference 27 for DLBCLs and GSE23026 for lymph nodes) in a cohort of DLBCLs and controls (lymph nodes) separating the analysis depending on the type, i.e. ABC-DLBCL and GC-DLBCL.

Black and light grey bars correspond to upregulated and downregulated miRNAs respectively from the RBL vs LCL comparison.

Supplementary Figure 1

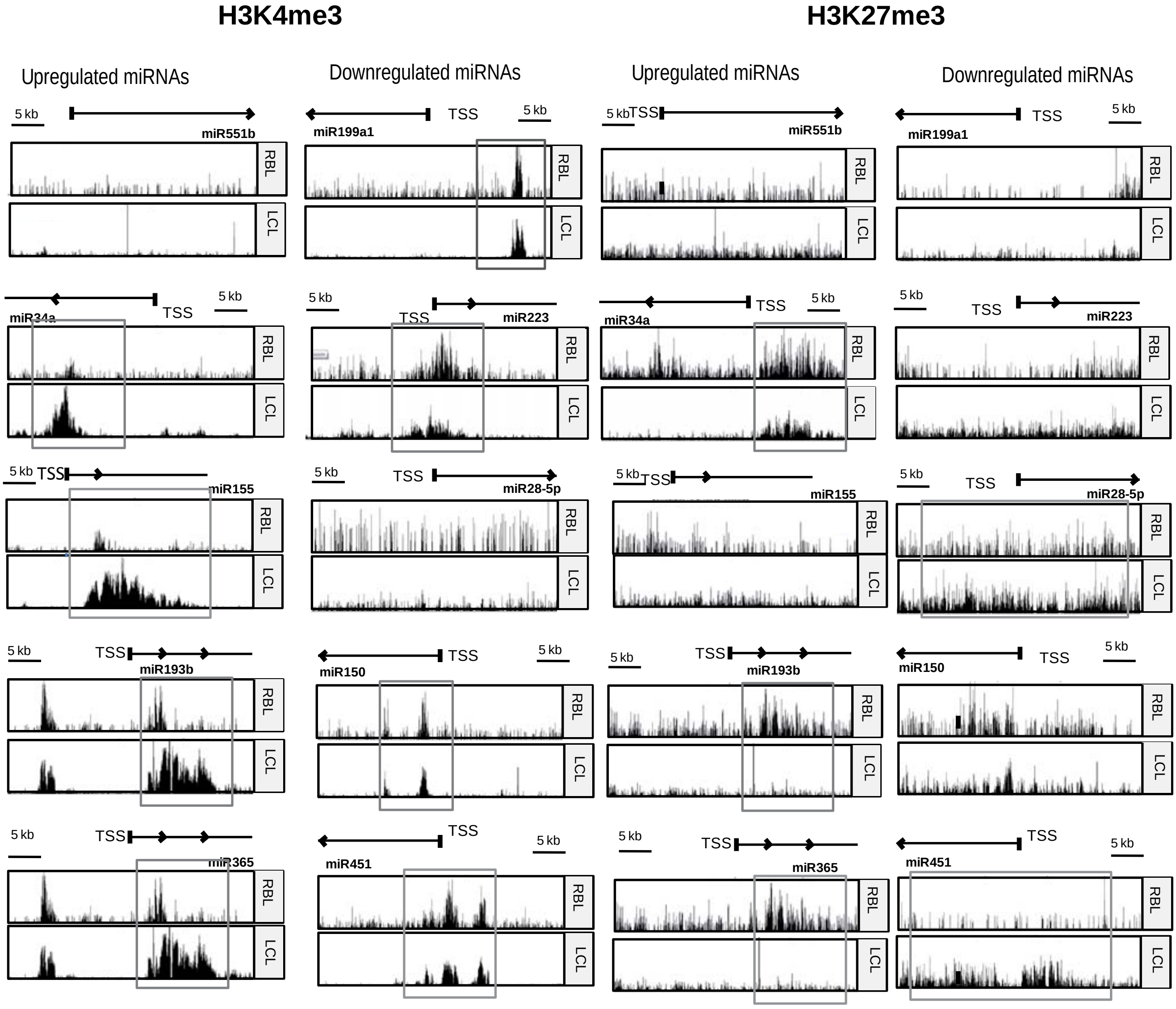

Supplementary figure 2

Binding motif	microRNA	Position from TSS
**NFkB p50 (M0051)**	miR-130b	275,284
	miR-34a	-372,-363
	miR-101	-471,-463
**NFkB p65 (M0052)**	miR-155	-279,-270
	miR-146a	-429,-420
	miR-146a	-105,-96
	miR-26b	-467,-458
	miR-26b	694,703
	miR-130b	-356,-347
	miR-101	-522,-513
**NFkB c-rel (M0053)**	miR-155	-279,-270
	miR-146a	-429,-420
	miR-146a	-105,-96
	miR-26b	-609,-600
	miR-26b	-467,-458
	miR-26b	694,703
	miR-28-5p	224,233
	miR-130b	-356,-347
	miR-627	-380,-371
	miR-627	-782,-773
	miR-708	-929,-920
	miR-101	-522,-513
	miR-18b	429,438
	miR-18b	-592,-583
**NFkB (M0054)**	miR-146a	-428,-419
	miR-146a	-105,-96
	miR-26b	-467,-458
	miR-223	786,795
	miR-130b	-356,-347
	miR-627	-379,-370
	miR-101	-522,-513
**NFkB (M0194)**	miR-146a	-107,-94
	miR-130b	-358,-347
**NFkB (M0208)**	miR-146a	-106,-95
	miR-130b	-357,-346
**NFkB p50:relA (M01224)**	miR-146a	-427,-416
**NFkB p52 (M01239)**	miR-146a	-105,-96
	miR-26b	-467,-458
	miR-26b	694,703
	miR-130b	-544,-535
	miR-130b	-356,-347
	miR-627	-380,-371
	miR-627	-782,-773
	miR-101	-522,-513

Supplementary Figure 3

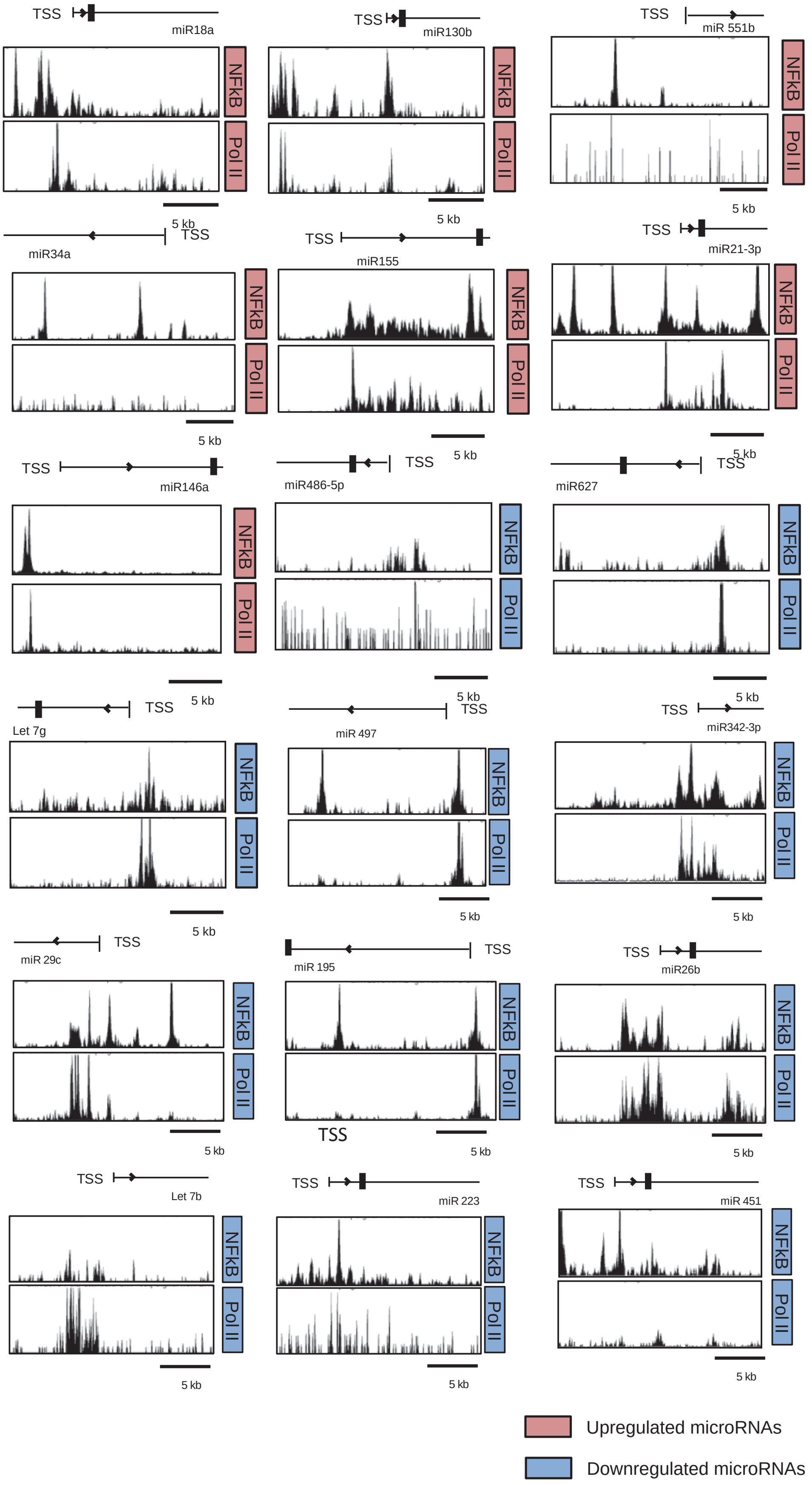

Supplementary Figure 4

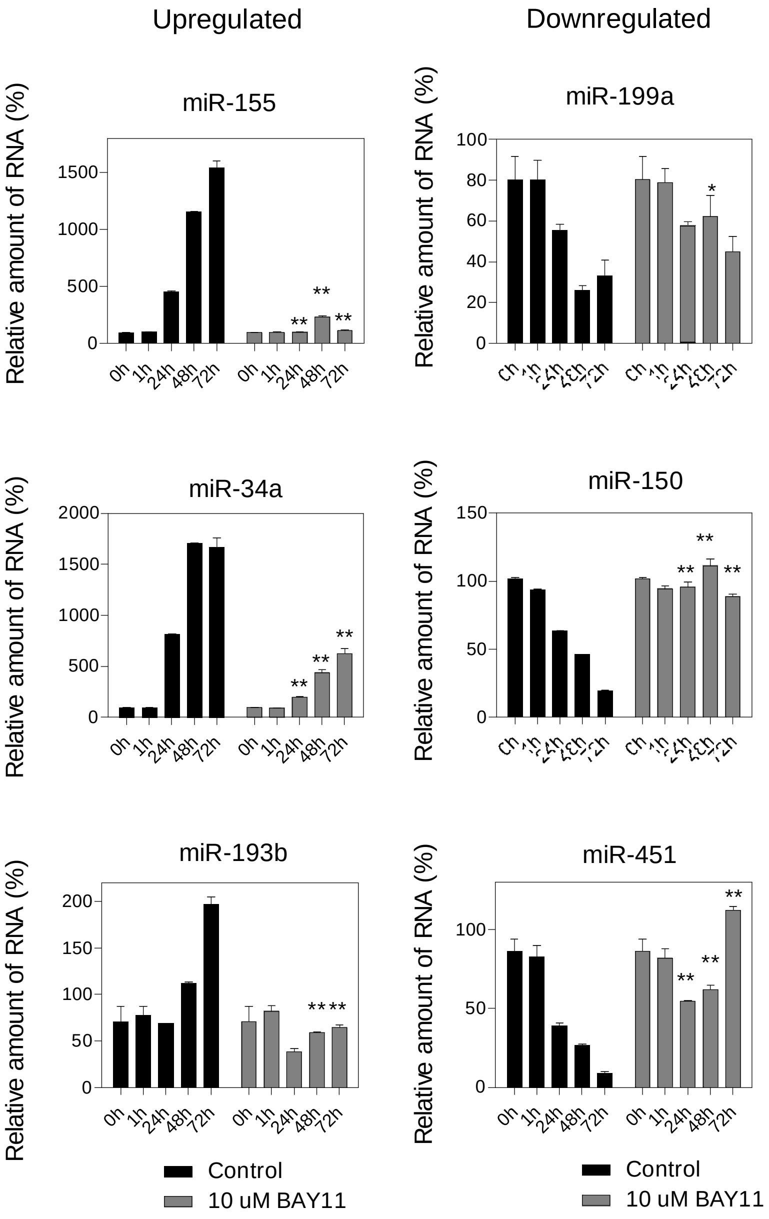

Supplementary figure 5

ABC-DLBCL

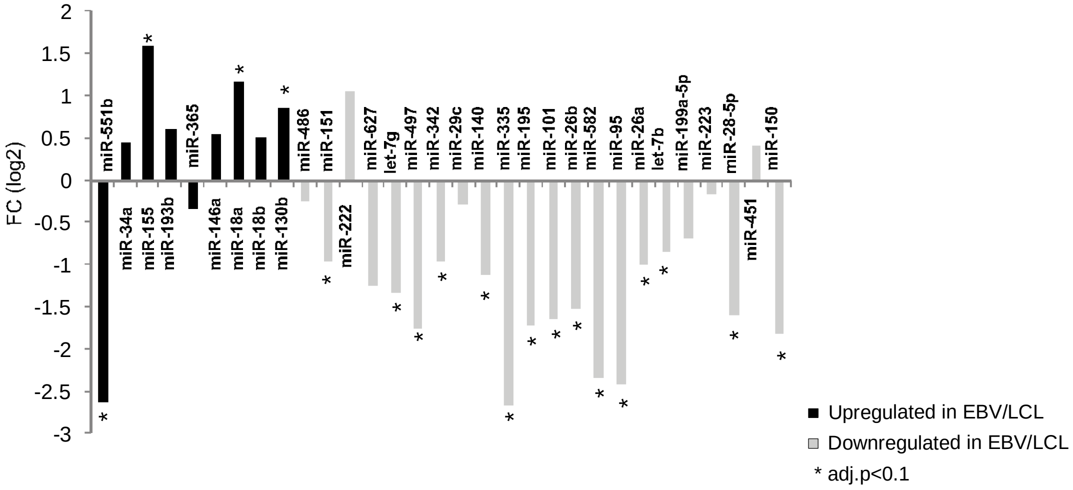

GC-DLBCL

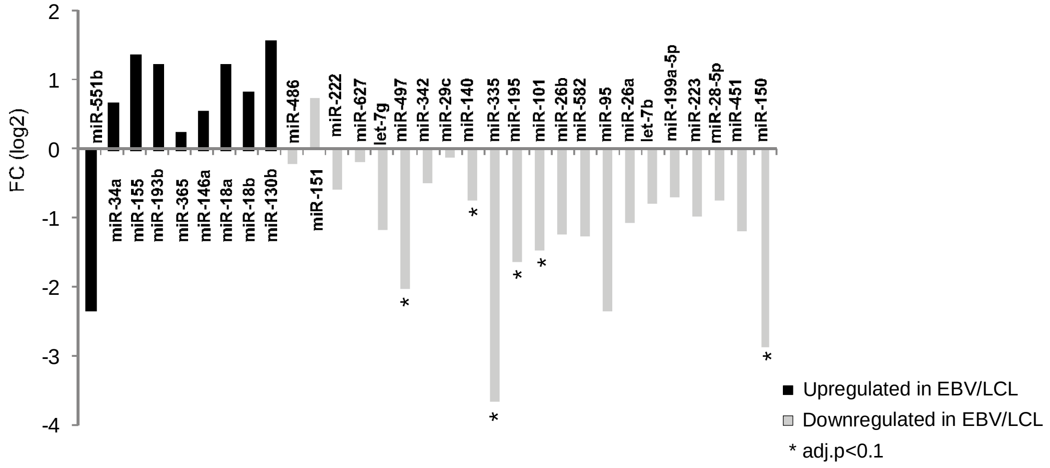

Supplement: SUPPLEMENTARY DATA [file supp_gku826_nar-01805-x-2014-File007.zip › NAR-01805-X-2014.R1 Suppl files/Supplementary_Material_Vento-Tormo.pdf]
