# Supplementary material for: NF-κB directly mediates epigenetic deregulation of common microRNAs in Epstein-Barr virus-mediated transformation of B-cells and in lymphomas
Source: Nucleic Acids Res. 2014 Sep 8;42(17):11025–39. doi: 10.1093/nar/gku826 (PMC4176189; doi:10.1093/nar/gku826)
Supplement: SUPPLEMENTARY DATA [file supp_gku826_nar-01805-x-2014-File007.zip › NAR-01805-X-2014.R1 Suppl files/Supplementary_Table_S2.doc]

**Supplementary Table S2.** Public ChIP-seq Data sources

| **Histone marks** | **Cell type** | **Data source** |
| --- | --- | --- |
| H3K4me3 | CD19  (RBL) | GEO accession GSE19465  [http://www.ncbi.nlm.nih.gov/geo/query/acc.cgi?acc=GSE19465]  **ChIP:**  GSM537622  GSM537632  **Input:**  GSM537620  <http://www.ncbi.nlm.nih.gov/geo/query/acc.cgi?acc=GSM537620> |
| H3K4me3 | GM12878 (LCL) | ENCODE (Broad Institute) [<http://hgdownload.cse.ucsc.edu/goldenPath/hg19/encodeDCC/wgEncodeBroadHistone>]  **ChIP:**  http://hgdownload.cse.ucsc.edu/goldenPath/hg19/encodeDCC/wgEncodeBroadHistone/wgEncodeBroadHistoneGm12878H3k4me3StdAlnRep1.bam  <http://hgdownload.cse.ucsc.edu/goldenPath/hg19/encodeDCC/wgEncodeBroadHistone/wgEncodeBroadHistoneGm12878H3k04me3StdAlnRep2V2.bam>  **Input:**  http://hgdownload.cse.ucsc.edu/goldenPath/hg19/encodeDCC/wgEncodeBroadHistone/wgEncodeBroadHistoneGm12878ControlStdAlnRep1.bam  http://hgdownload.cse.ucsc.edu/goldenPath/hg19/encodeDCC/wgEncodeBroadHistone/wgEncodeBroadHistoneGm12878ControlStdAlnRep2.bam |
|  |  |  |
| H3K27me3 | CD19  (RBL) | GEO accession GSE19465:  **ChIP:**  GSM537634  GSM537638  **Input:**  GSM537620 |
| H3K27me3 | GM12878 (LCL) | ENCODE (Broad Institute)  **ChIP:**  http://hgdownload.cse.ucsc.edu/goldenPath/hg19/encodeDCC/wgEncodeBroadHistone/wgEncodeBroadHistoneGm12878H3k27me3StdAlnRep1.bam  http://hgdownload.cse.ucsc.edu/goldenPath/hg19/encodeDCC/wgEncodeBroadHistone/wgEncodeBroadHistoneGm12878H3k27me3StdAlnRep2.bam  http://hgdownload.cse.ucsc.edu/goldenPath/hg19/encodeDCC/wgEncodeBroadHistone/wgEncodeBroadHistoneGm12878H3k27me3StdAlnRep3V2.bam  **Input:**  http://hgdownload.cse.ucsc.edu/goldenPath/hg19/encodeDCC/wgEncodeBroadHistone/wgEncodeBroadHistoneGm12878ControlStdAlnRep1.bam  http://hgdownload.cse.ucsc.edu/goldenPath/hg19/encodeDCC/wgEncodeBroadHistone/wgEncodeBroadHistoneGm12878ControlStdAlnRep2.bam |
|  |  |  |
| H3K9me3 | CD19  (RBL) | GEO accession GSE19465  **ChIP:**  GSM537635  GSM537654  **Input:**  GSM537620 |
| H3K9me3 | GM12878 (LCL) | ENCODE (Broad Institute)  **ChIP:**  http://hgdownload-test.cse.ucsc.edu/goldenPath/hg19/encodeDCC/wgEncodeBroadHistone/wgEncodeBroadHistoneGm12878H3k9me3StdAlnRep1.bam  http://hgdownload-test.cse.ucsc.edu/goldenPath/hg19/encodeDCC/wgEncodeBroadHistone/wgEncodeBroadHistoneGm12878H3k9me3StdAlnRep2.bam  **Input:**  http://hgdownload.cse.ucsc.edu/goldenPath/hg19/encodeDCC/wgEncodeBroadHistone/wgEncodeBroadHistoneGm12878ControlStdAlnRep1.bam  http://hgdownload.cse.ucsc.edu/goldenPath/hg19/encodeDCC/wgEncodeBroadHistone/wgEncodeBroadHistoneGm12878ControlStdAlnRep2.bam |
|  |  |  |
| p65 (NF-kB) |  | ENCODE  http://genome-euro.ucsc.edu/cgi-bin/hgTables?db=hg19&hgta_group=regulation&hgta_track=wgEncodeSydhTfbs&hgta_table=wgEncodeSydhTfbsGm12878NfkbTnfaIggrabSig&hgta_doSchema=describe+table+schema |
|  |  |  |
| Pol II |  | ENCODE  [http://genome-euro.ucsc.edu/cgi-bin/hgTables?db=hg19&hgta_group=regulation&hgta_track=wgEncodeSydhTfbs&hgta_table=wgEncodeSydhTfbsGm12878Pol2StdSig&hgta_doSchema=describe+table+schema](https://mail.idibell.cat/OWA/redir.aspx?C=99309b11c15c41dcb4ed2c175e9bd55c&URL=http%3A%2F%2Fgenome-euro.ucsc.edu%2Fcgi-bin%2FhgTables%3Fdb%3Dhg19%26hgta_group%3Dregulation%26hgta_track%3DwgEncodeSydhTfbs%26hgta_table%3DwgEncodeSydhTfbsGm12878Pol2StdSig%26hgta_doSchema%3Ddescribe%2Btable%2Bschema) |
